# Targeted Polymersome Delivery of a Stapled Peptide for Drugging the Tumor Protein p53:BCL-2-Family Axis in Diffuse Large B-Cell Lymphoma

**DOI:** 10.1021/acsnano.3c04112

**Published:** 2023-09-09

**Authors:** Mathew R. Schnorenberg, Katrina M. Hawley, Anika T. Thomas-Toth, Elyse A. Watkins, Yu Tian, Jeffrey M. Ting, Logan B. Leak, Isadora M. Kucera, Michal M. Raczy, Andrew L. Kung, Jeffrey A. Hubbell, Matthew V. Tirrell, James L. LaBelle

**Affiliations:** † Pritzker School of Molecular Engineering, University of Chicago, Chicago, Illinois 60637, United States; ‡ Department of Pediatrics, Section of Hematology/Oncology, University of Chicago, Chicago, Illinois 60637, United States; § Medical Scientist Training Program, Pritzker School of Medicine, University of Chicago, Chicago, Illinois 60637, United States; ∥ Department of Pediatrics, Memorial Sloan Kettering Cancer Center, New York, New York 10065, United States

**Keywords:** nanomedicine, toxicity, targeting, stapled peptide, DLBCL, apoptosis

## Abstract

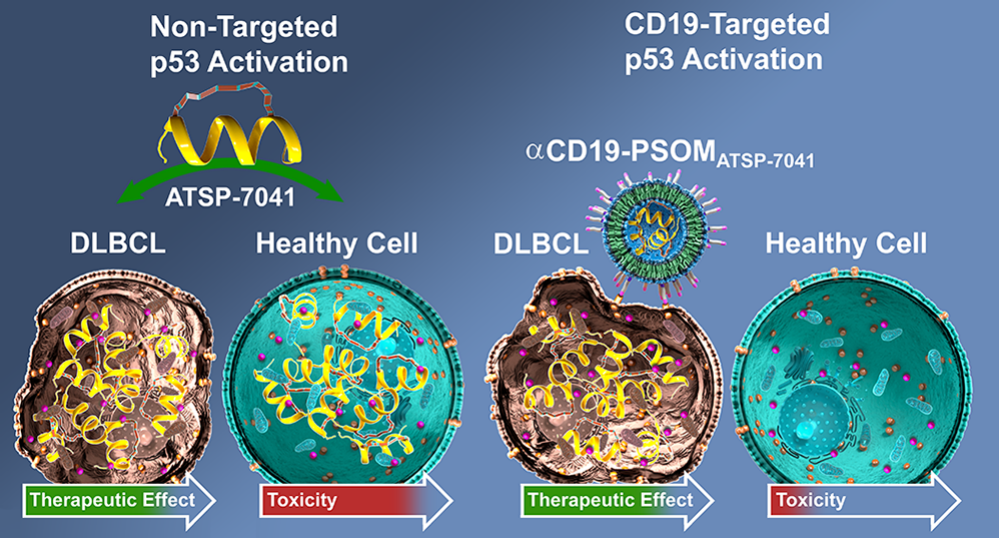

Diffuse large B-cell lymphoma (DLBCL) remains a formidable diagnosis in need of new treatment paradigms. In this work, we elucidated an opportunity for therapeutic synergy in DLBCL by reactivating tumor protein p53 with a stapled peptide, ATSP-7041, thereby priming cells for apoptosis and enhancing their sensitivity to BCL-2 family modulation with a BH3-mimetic, ABT-263 (navitoclax). While this combination was highly effective at activating apoptosis in DLBCL *in vitro*, it was highly toxic *in vivo*, resulting in a prohibitively narrow therapeutic window. We, therefore, developed a targeted nanomedicine delivery platform to maintain the therapeutic potency of this combination while minimizing its toxicity via packaging and targeted delivery of a stapled peptide. We developed a CD19-targeted polymersome using block copolymers of poly(ethylene glycol) disulfide linked to poly(propylene sulfide) (PEG-SS-PPS) for ATSP-7041 delivery into DLBCL cells. Intracellular delivery was optimized *in vitro* and validated *in vivo* by using an aggressive human DLBCL xenograft model. Targeted delivery of ATSP-7041 unlocked the ability to systemically cotreat with ABT-263, resulting in delayed tumor growth, prolonged survival, and no overt toxicity. This work demonstrates a proof-of-concept for antigen-specific targeting of polymersome nanomedicines, targeted delivery of a stapled peptide *in vivo*, and synergistic dual intrinsic apoptotic therapy against DLBCL via direct p53 reactivation and BCL-2 family modulation.

DLBCL is the most common form of non-Hodgkin lymphoma, and 60% of patients are initially diagnosed with advanced-stage III or IV disease.^1,2^ One in three treated patients with DLBCL will not survive five years, and half will not survive ten years.^2^ One of the reasons for DLBCL’s resistance to treatment is its ability to inactivate intrinsic apoptotic pathways where cell fate decisions occur through well-defined and highly specific protein-protein interactions (PPIs) between the pro- and antiapoptotic BCL-2 family members.^3−7^ A promising paradigm in DLBCL treatment is the use of BH3-mimetics, small molecules designed to specifically block these PPIs and inhibit BCL-2 family antiapoptotic sequestration of proapoptotic members to reactivate apoptosis.^8^ Venetoclax, or ABT-199, was designed to inhibit BCL-2 specifically and was the first FDA-approved example of such a drug.^9,10^ However, for relapsed and refractory DLBCL, venetoclax has had minimal antitumor effect despite 97% of patients experiencing treatment-related adverse events.^9^ One reason for this failure is that rather than relying primarily on a single antiapoptotic protein (e.g., BCL-2) to prevent cell death, DLBCL is heterogeneous in its cell death evasion by upregulating multiple antiapoptotic proteins and downregulating multiple proapoptotic proteins,^5,10,11^ making it particularly difficult to overcome the apoptotic blockade. Navitoclax, or ABT-263, is a precursor to venetoclax that targets multiple BCL-2 family antiapoptotic proteins (i.e., BCL-2, BCL-W, and BCL-X_L_) and has shown preclinical potency against several cancers, including DLBCL.^12^ However, navitoclax causes dose-limiting thrombocytopenia in patients due to platelet dependence on BCL-X_L_ for survival.^13−16^ Thus, the therapeutic window is currently prohibitively narrow for directly reactivating apoptosis and overcoming chemoresistance in many patients with DLBCL.

However, one opportunity for sensitizing DLBCL to apoptosis is through the therapeutic activation of wild-type (WT) p53.^17^ Among other roles, p53 primes cells for apoptosis by transcriptionally upregulating proapoptotic and downregulating antiapoptotic BCL-2 family members.^18^ However, WTp53 inactivation by its inhibitory binding partners, HDM2 and HDMX, is common in DLBCL and correlates with inferior survival.^19,20^ While preclinical p53 reactivation in DLBCL has been shown to overcome BCL-2 overexpression, single-agent small-molecule therapies that reactivate p53 have been clinically underwhelming, and preclinical combination studies with BH3-mimetics have not led to clinical translation in large part due to toxicity and the inability to target MDMX.^21−23^ DLBCL represents an opportunity for sensitization to BH3-mimetics via p53 reactivation, as over 80% of DLBCL patients have disease harboring WTp53 inactivated by upregulation of p53’s inhibitory binding partners HDM2 and HDMX.^24^ Unlike current small-molecule activators of p53, a hydrocarbon-stapled peptide, ALRN-6924, and its preclinical predecessor, ATSP-7041, potently inhibits both HDM2 and HDMX. ATSP-7041 has shown promising antitumor effects in multiple preclinical models,^25−27^ but ALRN-6924 has not yet been translated clinically.^25−27^ One of the most significant challenges for using this class of therapeutics in patients is their lack of cellular specificity and minimization of significant side-effects of p53 activation in normal cells, especially when combined with other chemotherapies, necessitating a more targeted delivery approach.^28^

In this work, we hypothesized that p53 reactivation using ATSP-7041 would prime DLBCL for apoptosis via the BCL-2 family of proteins and sensitize it to therapeutic cell death by ABT-263. Indeed, this was a potent combination *in vitro* but caused significant toxicity *in vivo.* To widen the therapeutic window between the antitumor and toxic side effects, we designed a targeted nanoparticle delivery system to deliver ATSP-7041 specifically into DLBCL cells, thus maintaining the therapeutic synergy while enabling significantly lower and less toxic dosing of ABT-263. This work demonstrates the packaging of a hydrocarbon-stapled peptide and the tolerable concomitant targeting of synergistic intrinsic apoptotic pathways in DLBCL. We believe that such nanoparticle delivery of stapled peptides could meaningfully expand the use of this promising drug class against a myriad of diseases.

## Results and Discussion

### Simultaneous p53 Reactivation and BCL-2 Family Inhibition Are Synergistic against DLBCL *In Vitro* but Toxic *In Vivo*

**Figure 1:**
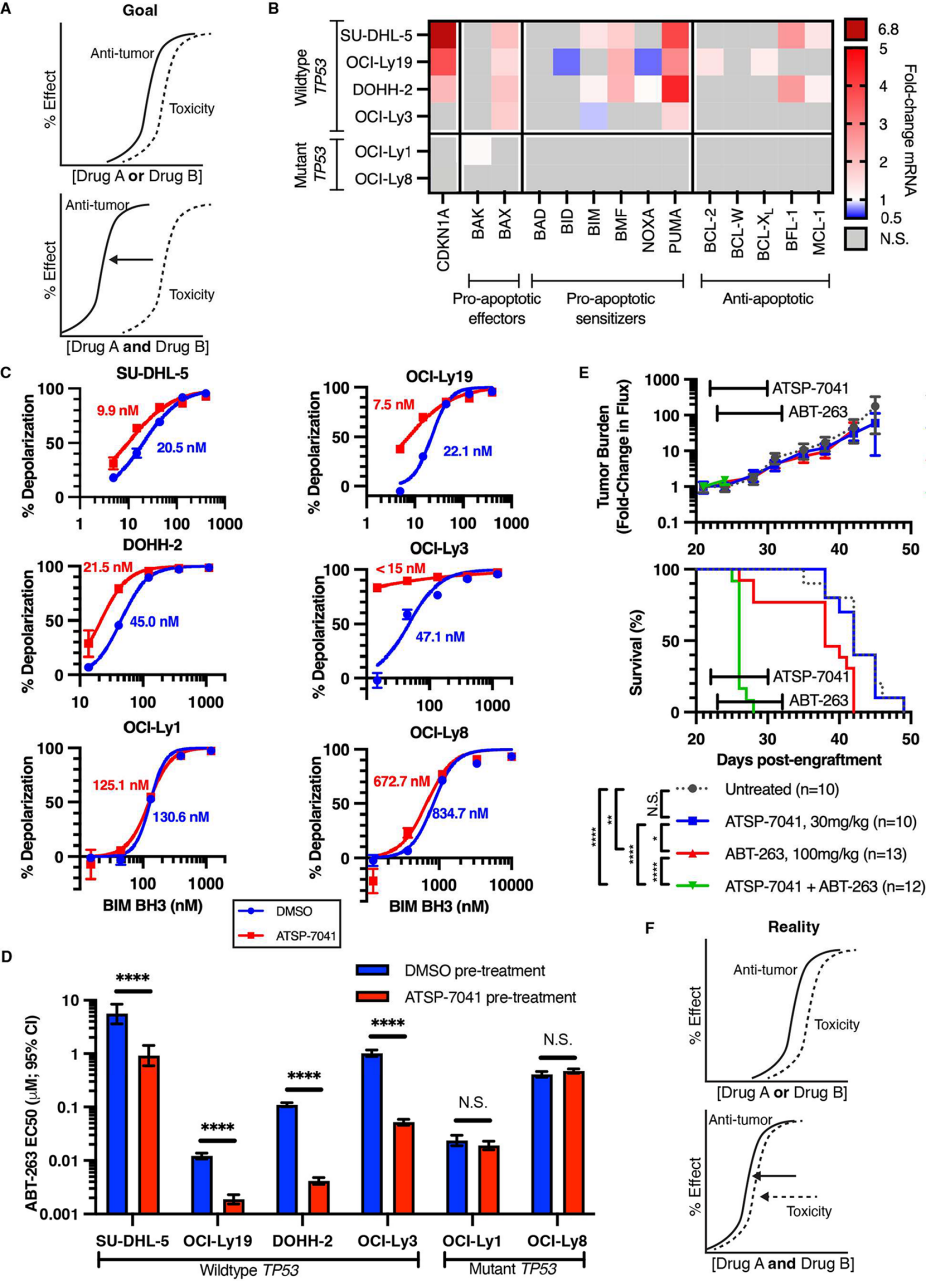
p53 reactivation and BCL-2-family inhibition are synergistic against DLBCL *in vitro* but toxic *in vivo*. (A) The ideal dose–effect relationship for antitumor and toxicity effects when combining two antitumor drugs to widen the therapeutic window. (B) Changes in mRNA expression of the BCL-2 family in response to p53 reactivation by ATSP-7041. Plotted values are the mean of biological triplicates. Changes were deemed significant via a one-sample *t* test (*H*_0_: ΔΔCT = 0; *p* < 0.05; N.S. = not significant). (C) Sensitivity to mitochondrial depolarization in DLBCL cell lines after p53 reactivation by ATSP-7041. Plotted values are the mean of triplicates ± SEM and fitted to a normalized nonlinear regression with variable slope. (D) Cell death sensitivity of DLBCL cell lines to ABT-263 with or without pretreatment with ATSP-7041. Plotted values are the EC_50_ and 95% confidence interval calculated from dose titrations in duplicate fitted to a normalized nonlinear regression with a variable slope. Pretreatment conditions within each cell line were compared via extra sum-of-squares *F* test. *H*_0_ = EC50 is identical between dose titration curves. **** *p* < 0.0001, N.S. *p* > 0.05. (E) Tumor burden and survival of mice with disseminated OCI-Ly19-Luc treated with ATSP-7041 (blue), ABT-263 (red), both (green), or neither (gray). * *p* < 0.05, ** *p* < 0.01, *** *p* < 0.001, **** *p* < 0.0001, N.S. = not significant. (F) The theoretical dose–effect relationship when combining two antitumor drugs enhances both the antitumor effect and the toxicity effect, resulting in a narrow therapeutic window.

We first sought a therapeutic combination that would be synergistic in a wide variety of DLBCL subtypes with and without BCL-2 overexpression.^29,30^ We hypothesized that reactivating p53 using ATSP-7041 would sensitize DLBCL to cell death by ABT-263 at lower, less toxic doses, regardless of their BCL-2 expression and BCL-2 family-mediated apoptotic resistance, and result in a widened therapeutic window (Figure 1A). To determine if ATSP-7041 would prime cells to die via p53-mediated transcriptional regulation, we treated various human DLBCL cell lines with ATSP-7041 and measured resultant BCL-2 family mRNA changes (Figures 1B, S1, S2). In DLBCL with WTp53 (i.e., SU-DHL-5, OCI-Ly19, DOHH-2, and OCI-Ly3), ATSP-7041 treatment reactivated p53, as evidenced by the upregulation of canonical p53 transcriptional targets, including *CDKN1A*. WTp53 activation also resulted in proapoptotic BCL-2 family gene expression changes, such as upregulation of *PUMA* and *BAX*. Compensatory antiapoptotic gene expression changes, as have been shown to accompany BCL-2 proapoptotic alterations,^31,32^ also occurred (e.g., *BFL-1*). Next, we measured whether these mRNA changes corresponded to functional apoptotic priming, where the overall sensitivity to mitochondrial-mediated cell death was measured using BH3 priming. In this assay, cells are treated with a peptide mimetic of the BH3 domain of BIM, the most potent BH3-only proapoptotic BCL-2 family member, which activates outer mitochondrial membrane permeabilization (MOMP) and mitochondrial depolarization. The amount of BIM BH3 peptide required to depolarize the mitochondria is inversely related to how primed the cells are for apoptosis (i.e., if less BIM BH3 peptide is required to depolarize the mitochondria, the cells are more primed for apoptosis). In response to pretreatment with ATSP-7041, DLBCL with WTp53 became more sensitive to mitochondrial depolarization (Figure 1C), confirming that therapeutic p53 activation primed DLBCL for apoptosis. This ATSP-7041-mediated increased priming corresponded with significantly greater sensitivity to ABT-263 (Figures 1D, S3). However, when we sought to harness this therapeutic combination *in vivo*, the combination of ATSP-7041 and ABT-263 was highly toxic, precluding any measurable antitumor effect (Figure 1E). Therefore, while p53 reactivation primed a broad set of DLBCL cell lines for therapeutic cell death by BCL-2 family inhibition, it also markedly enhanced *in vivo* toxicity, thus shifting but not widening their combined therapeutic window (Figure 1F).

### Design of CD19-Targeted Polymersomes to Deliver ATSP-7041 to DLBCL Cells, Prime Them for Cell Death by ABT-263, and Widen the Therapeutic Window

**Figure 2:**
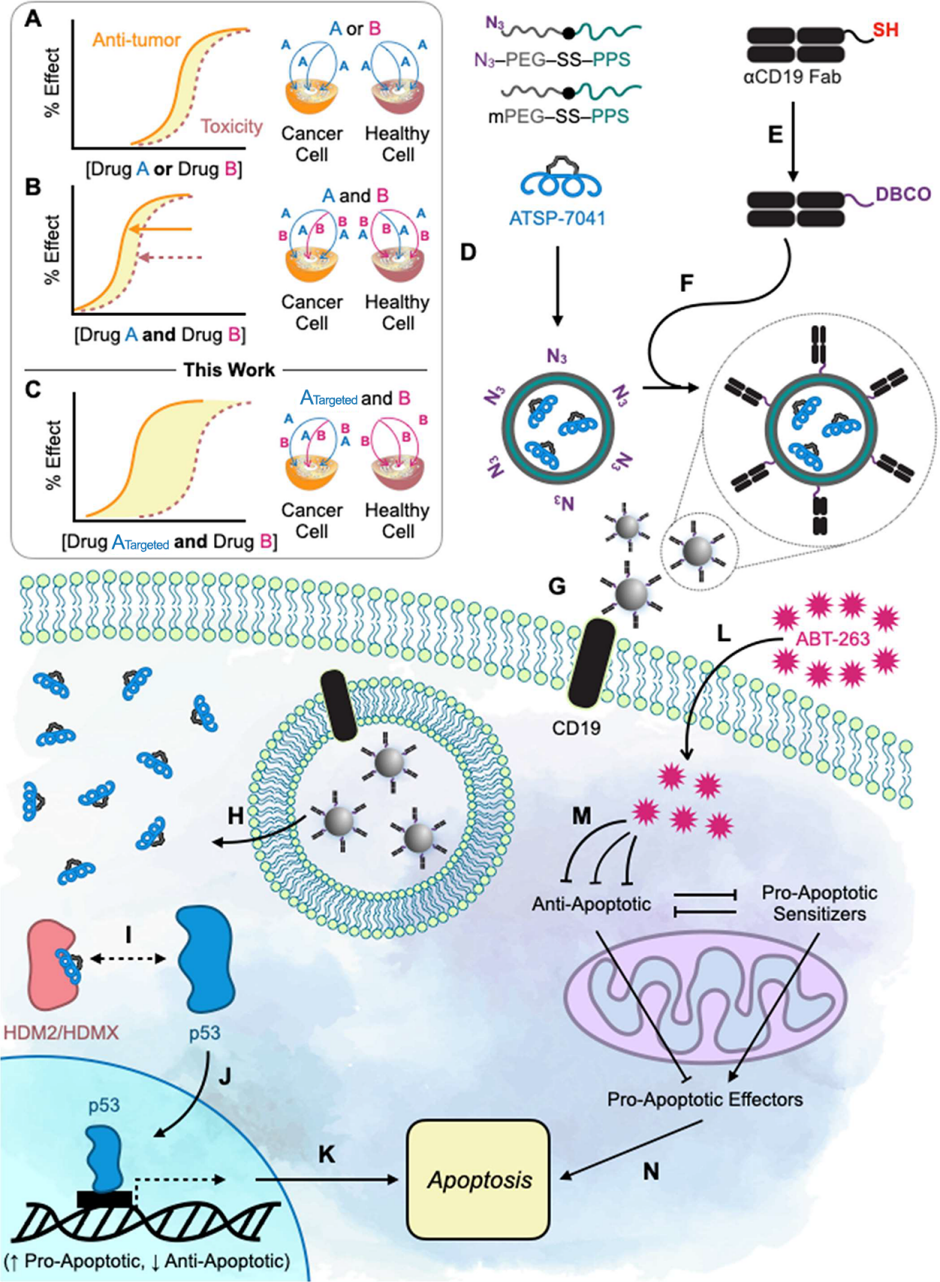
Vision, design, and experimental summary of CD19-targeted polymersomes to deliver ATSP-7041 to DLBCL cells, prime them for cell death by ABT-263, and widen their combined therapeutic window. (A) Each drug has a dose–effect relationship for antitumor effect and toxicity, and the therapeutic window lies between them. (B) When two drugs are combined, both antitumor effects and toxicity can be achieved at lower doses. (C) The therapeutic window for a drug combination can be widened when one drug is targeted specifically to cancer cells. (D) Stapled-peptide ATSP-7041 is stably encapsulated in the PEG-SS-PPS polymersomes. (E) Recombinant αCD19 Fabs are functionalized with a site-specific DBCO click chemistry handle. (F) The polymersomes are decorated with αCD19 Fabs, and the targeted polymersomes (αCD19-PSOMs) are purified. (G) αCD19-PSOMs bind CD19 on DLBCL cells and are endocytosed. (H) The ATSP-7041 cargo is released from the endosome-responsive polymersomes and into the cytoplasm. (I) ATSP-7041 binds HDM2 and HDMX to release p53, which (J) reactivates its tumor suppressor function, allowing it to translocate to the nucleus, upregulate proapoptotic genes, downregulate antiapoptotic genes, and (K) prime DLBCL cells for apoptosis. (L) Systemic ABT-263 (M) inhibits antiapoptotic proteins in the BCL-2 family to (N) induce apoptosis.

We next sought to mitigate the *in vivo* toxicity while harnessing the therapeutic combination of p53 reactivation and BCL-2 family modulation (Figure 2A,B). The goal was to target the delivery of ATSP-7041 to prime DLBCL for ABT-263-mediated apoptotic reactivation while leaving nonmalignant cells unprimed (Figure 2C). Additionally, while hydrocarbon-stapled peptides are potent PPI inhibitors *ex vivo*, their *in vivo* pharmaceutical properties and cellular delivery remain significant challenges to their therapeutic translation.^33^ In this regard, we hypothesized that their *in vivo* efficacy could be increased by packaging and directing them to tumor cells.

To accomplish this, we envisioned a multifunctional platform that could encapsulate hydrocarbon stapled peptides, was stable in serum, could be targeted to and endocytosed by DLBCL, would release its cargo intracellularly, was itself nontoxic, and was scalable in its production. We believed that the amphiphilic block copolymer, PEG-SS-PPS, previously shown to form polymersomes (PSOMs), would have the ideal characteristics of a nanocarrier for adaptation to this application. Our strategy was as follows: PEG-SS-PPS block copolymers would be self-assembled into highly stable PSOMs either through thin-film assembly or, perhaps more scalable, flash nanoprecipitation (Figure 2D).^34−36^ Due to its amphiphilicity, PEG-PPS (without a disulfide bond) and PEG-SS-PPS (with a disulfide bond) have been used to successfully encapsulate both hydrophilic and hydrophobic cargoes into nanomaterials,^37−41^ and we hypothesized that these could also encapsulate amphiphilic ATSP-7041 into PSOMs (“PSOM_ATSP-7041_”). Synthesis of these polymers with bioorthogonal click chemistry functional groups (e.g., N_3_) allows linkage of a DLBCL-relevant targeting element to their surface.^42^ To accomplish this, a recombinant F(ab) antibody fragment (“Fab”) against CD19 (“αCD19 Fab”), an endocytic B-cell surface marker,^43−46^ was designed to take advantage of the fact that CD19 expression is rarely lost in DLBCL, and not expressed in hematopoietic stem cells.^47^ αCD19 Fab with a C-terminal cysteine linker (“αCD19-SH”) was functionalized for bioorthogonal click chemistry (“αCD19-DBCO”; Figure 2E) to decorate the surface of intact PSOMs (“αCD19-PSOM”; Figure 2F). CD19-targeted PSOMs would then theoretically retain ATSP-7041 in circulation until binding to CD19-expressing DLBCL cells (Figure 2G), where it would be endocytosed. The redox-responsive disulfide bond between the hydrophilic PEG and hydrophobic PPS domains could then be reduced in endosomes to facilitate cargo release and intracellular accumulation (Figure 2H).^37^ Meanwhile, oxidation of the PPS backbone within phagolysosomes would similarly release the stapled-peptide cargo and convert the block copolymers into nontoxic hydrophilic unimers.^39,48,49^ Both the reduction-triggered release and oxidation-triggered release mechanisms have been thoroughly characterized.^37,39,48^ Once inside DLBCL cells, ATSP-7041 would release p53 from sequestration by HDM2 and HDMX, allowing p53 to translocate to the nucleus, execute its transcriptional function, and prime DLBCL cells for apoptosis (Figure 2I–K).^18,25^ Lower, less toxic doses of systemic ABT-263 treatment (Figure 2L) could then inhibit key BCL-2 family antiapoptotic proteins (Figure 2M) and activate apoptosis (Figure 2N) in these same cells.

### PEG-SS-PPS Polymersome Assembly, Characterization, and Stapled-Peptide Encapsulation

**Figure 3:**
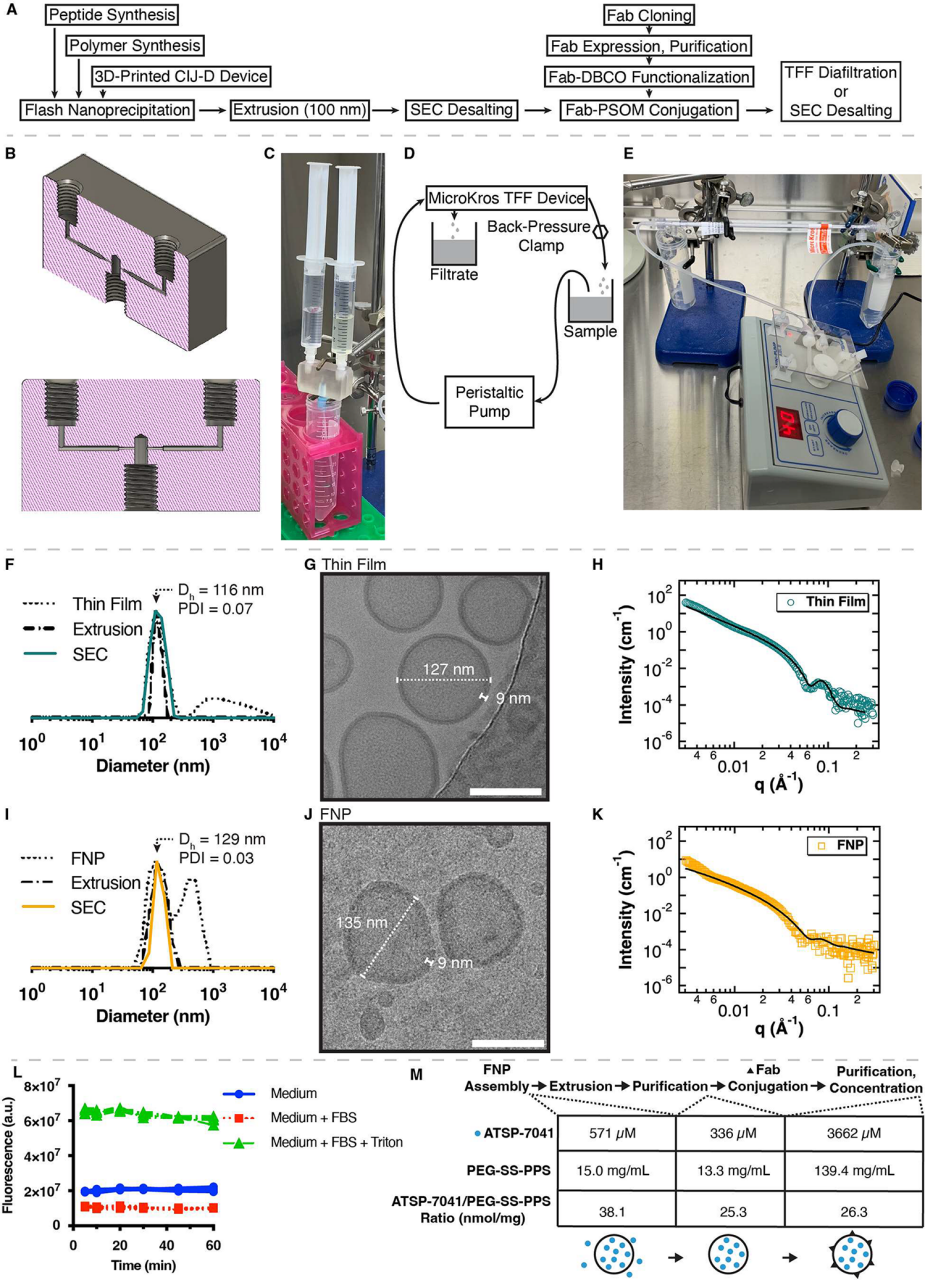
PEG-SS-PPS polymersome assembly, characterization, stability in serum, and ATSP-7041 encapsulation. (A) Production workflow for Fab-targeted polymersomes encapsulating stapled peptides. (B) Computer-aided design of a 3D-printed CIJ-D device for flash nanoprecipitation. (C) Photo of the scalable flash nanoprecipitation hardware. (D,E) Design (D) and photo (E) of a system for scalable, cost-effective, sterile, and semiautomated diafiltration for purification and concentration of Fab-polymersomes using a peristaltic pump and MicroKros TFF device. (F–K) PEG-SS-PPS formed uniform polymersomes following assembly, extrusion, and SEC desalting. (F,I) DLS measurements of empty polymersomes formed by (F) a thin-film method (“Thin Film”) or by (I) flash nanoprecipitation (“FNP”), followed by extrusion through a 100 nm pore-size membrane (“Extrusion”) and desalting into PBS (“SEC”). Plotted are the intensity-scaled size distributions from the Regularization fit method. *D*_h_ and PDI are listed for the final SEC-purified samples. (G,J) Cryo-TEM images confirm that the polymersomes are uniform hollow spheres with diameters and bilayer thicknesses corresponding to DLS and SAXS measurements, whether formed by a (G) thin film or (J) FNP. Scale bars are 100 nm. (H,K) SAXS data (open circles and squares) fit well to a hollow sphere model (solid line) for both thin-film and FNP-formed polymersomes. (L) Polymersomes encapsulating a self-quenching calcein solution were diluted into various solutions at equal concentrations, and fluorescence dequenching due to polymersome disruption was monitored for 1 h at 37 °C. Data plotted are individual quadruplicates, each background-subtracted against samples in which an equivalent volume of PBS was added instead of polymersomes. (M) Efficiency of peptide retention in the polymersomes was measured by monitoring the ratio of the ATSP-7041 concentration to the PEG-SS-PPS concentration at the indicated points in the assembly process. Shown are measurements for the large-scale formulation concentrated for IV use. A cartoon depicts a polymersome (black circle), peptides (blue circles), and Fabs (black triangles).

To assemble αCD19-PSOMs (Figure 3A), synthesis and characterization of the individual components (i.e., ATSP-7041, PPS-PDS, mPEG-SS-PPS, N_3_-PEG-SS-PPS, αCD19-Fab, negative control αOspA-Fab) were first performed (Figures S4–S10). A 3D-printed flash nanoprecipitation (FNP) confined impingement jets with dilution (CIJ-D) device was additionally developed (Figure 3B, File S1) to rapidly form PSOMs at a wide range of production scales (Figure 3C), building on what has been reported previously with PEG-PPS.^35,36^ Precursor PSOMs were extruded through a 100 nm membrane to ensure final size uniformity and desalted (“SEC desalting”) to remove residual organic solvent and nonencapsulated cargo. Finally, Fabs were expressed, functionalized with a DBCO click chemistry handle (“Fab-DBCO”), and attached to the surface of the PSOMs (“Fab-PSOM”), and this was followed by the removal of nonconjugated Fab. Fab-PSOM samples were then purified using a tangential flow filtration (TFF) system (Figure 3D,E).

This modular assembly strategy produced PSOMs with various cargoes and targeting moieties. FNP-generated PSOMs were indistinguishable in size, dispersity, and shape from those made by the more traditional but less scalable thin-film assembly strategy (Figure 3F–K). In addition, the resulting PSOMs were highly stable in the presence of serum without calcein dequenching (Figure 3L). Then, ATSP-7041 was encapsulated in PEG-SS-PPS PSOMs via FNP assembly (Figure 3M). To monitor retention of ATSP-7041 within the PSOMs, the ratio of ATSP-7041 to PEG-SS-PPS was monitored at multiple points in the assembly process for the large-scale formulation concentrated for IV use. When the PSOMs were purified, the nonencapsulated ATSP-7041 was removed and 66% remained (ATSP-7041/PEG-SS-PPS ratio 38.1 → 25.3). After the targeting Fab was attached, the PSOMs were again purified and then concentrated for IV use, and no peptide was lost in this final purification step (ATSP-7041/PEG-SS-PPS ratio 25.3 → 26.3), suggesting complete retention inside the PSOMs. In this representative sample, the final drug loading was 4.4% (w/w) (peptide/(peptide + polymer)).

When this assembly strategy was attempted with a similar peptide without a hydrocarbon staple (p53_(14–29)_), no measurable peptide was detected by LCMS in the polymersomes after the first purification step. We hypothesize that the hydrocarbon staple plays an unidentified but important role in the encapsulation of ATSP-7041 in the PEG-SS-PPS PSOMs. However, there was no discernible difference in the size, shape, or bilayer thickness of empty PSOMs compared to PSOMs encapsulating ATSP-7041 (Figure S8).

Resultant PSOMs were thereafter reproducibly assembled with various cargoes (i.e., ATSP-7041, calcein) to evaluate their targeting, intracellular uptake, and killing of DLBCL *in vitro* and *in vivo*.

### Characterization of Targeted αCD19-PSOMs and Delivery into DLBCL *In Vitro*

Fab-targeting PSOMs encapsulating calcein (″αCD19-PSOM_calcein_”) were developed to optimize PSOM binding to and internalization into DLBCL. A Fab targeting human CD19 on DLBCL (i.e., αCD19) was engineered with and without a C-terminal hydrophilic linker with a terminal cysteine residue for site-specific attachment to polymersomes (Figure S9). The C-terminal additions did not affect binding to CD19^+^ DLBCL (Figure S10). To evaluate for any nonspecific binding, a control Fab was generated with identical constant regions and C-terminal linker but variable regions targeting *Borrelia burgdorferi* outer surface protein A (i.e., αOspA). This control Fab also did not bind to CD19^+^ DLBCL (Figure S10), confirming that the αCD19 Fab binds DLBCL specifically via CD19.

**Figure 4:**
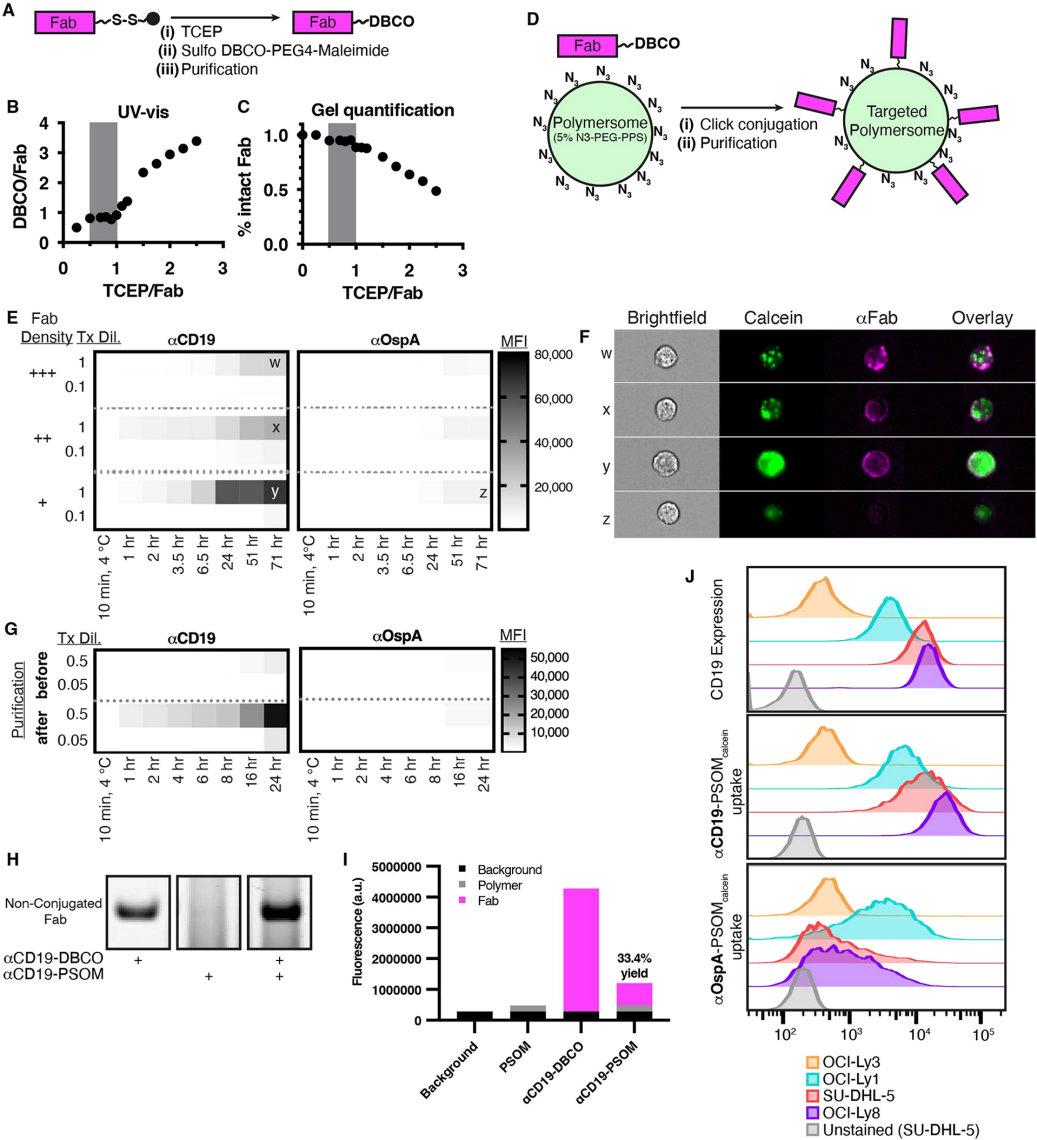
CD19-targeted polymersomes deliver a model fluorescent cargo into DLBCL cells *in vitro*. (A) Disulfide-capped Fabs were (i) reduced, (ii) DBCO-functionalized, and (iii) purified. (B,C) Fabs reduction was tested with a range of TCEP stoichiometries. (B) The DBCO/Fab ratio was determined by UV–vis absorbance, and (C) the percent of intact Fab was quantified by a Coomassie-stained SDS-PAGE gel. *Y*-values were normalized to the ratio of intact, unimeric Fab before the reaction (in this case, 80%), as measured by SDS-PAGE. The optimized TCEP/Fab ratio range is indicated by gray shading. (D) Fab-targeted polymersomes were generated by (i) a click chemistry reaction and (ii) purified to remove any nonconjugated Fab. (E) CD19-targeting enhances polymersome delivery of calcein into DLBCL cells: +++ = 1% theoretical Fab density, ++ = 0.5% theoretical Fab density, + = 0.1% theoretical Fab density. OCI-Ly8 cells were treated as indicated, and calcein uptake was measured by flow cytometry. (F) To visualize intracellular and extracellular localization, representative samples were taken from the latest time point in (E) and visualized using ImageStream single-cell imaging. Representative single cells from the indicated treatment conditions (“w”, “x”, “y”, and “z” in panel (E)) were imaged for the following: cellular integrity (brightfield), calcein (green), and anti-Fab extracellular staining (magenta). The last column represents an overlay of calcein and anti-Fab staining. (G) SU-DHL-5 cells were treated similarly as in (E), with polymersomes either before the removal of nonconjugated Fab (“before” purification) or with pure polymersomes (“after” purification). (H) Coomassie-stained SDS-PAGE confirmed the disappearance of the Fab-DBCO band after conjugation and purification. Each lane was loaded with identical amounts of conjugated Fab. (I) Protein quantification of the amount of Fab in the final samples (“αCD19-PSOM”), background-subtracted from blank (“Background”) and empty polymersome (“PSOM”) samples. (J) CD19-specific uptake was correlated with CD19 expression on DLBCL cell lines. Cells were either stained with fluorescent αCD19 IgG or treated with αCD19-PSOM_calcein_ or αOspA-PSOM_calcein_ for 24 h. An unstained, untreated sample of SU-DHL-5 is shown for comparison.

The exposed cysteine on each Fab was initially nonreactive, presumably in a mixed disulfide with cysteine or glutathione, as previously demonstrated in a similar application.^50^ The exposed cysteine, therefore, required TCEP reduction before the addition of a heterobifunctional linker (i.e., sulfo DBCO-PEG4-maleimide) to generate a DBCO click chemistry group (Figure 4A). A broad range of TCEP/Fab ratios was tested to functionalize the exposed, C-terminal cysteine without reducing native disulfide bonds within the Fab (Figures 4B,C, S11). With less than 0.5 equivalents of TCEP, the DBCO/Fab ratio was less than 1, indicating incomplete functionalization (Figure 4B). With increasing equivalents of TCEP greater than 1, the DBCO/Fab ratio quickly increased above 1 while the percentage of intact Fab decreased, indicating native disulfide reduction and DBCO functionalization (Figure 4B,C). From these data, we determined that the reliable range of TCEP needed to reduce the terminal cysteine linker and functionalize it with DBCO was between 0.5 and 1 equivalents, as represented by the gray bar in each graph (Figure 4B,C). Using this optimized DBCO-functionalization approach, we reliably generated αCD19-DBCO and αOspA-DBCO with a DBCO/Fab ratio of ∼1. Fab-DBCO was mixed with PSOMs containing 5% N_3_-PEG-SS-PPS, and resultant Fab-PSOMs were purified to remove residual nonconjugated Fab (Figure 4D). αCD19-PSOM_calcein_ and αOspA-PSOM_calcein_ were then generated with a wide range of surface-bound Fab densities to determine whether an optimal Fab:PSOM ratio existed for efficient ligand binding and cellular internalization. We found that the degree of Fab surface density greatly affected the intracellular delivery of calcein (Figures 4E,F, S12). Interestingly, a higher Fab density on αCD19-PSOM_calcein_ resulted in diminished calcein binding and internalization by DLBCL cells as measured by flow cytometry and did not lead to more intracellular accumulation of calcein over time. Based on these data, the lowest tested Fab density (as indicated by “+” in Figure 4E; 0.1% theoretical polymer surface functionalization) maximized PSOM binding and cargo internalization into DLBCL and was used in subsequent experiments. Intracellular calcein accumulation was also dependent on time and concentration. DLBCL cells were treated from 0–71 h, and while most calcein accumulation occurred in the first 24 h, there was additional uptake as late as 71 h (Figures 4E,F, S12). Uptake was also concentration-dependent, as 10-fold diluted treatments resulted in decreased uptake (Figures 4E, S12), and no concentration was achieved at which uptake plateaued (Figure S13). The cellular accumulation was confirmed to be intracellular using ImageStream (Figure 4F).

The final purification step of removing nonconjugated Fab proved critical during *in vitro* testing because, if omitted, nonconjugated Fab in the treatment mixture blocked antigen-specific binding and cellular uptake of the PSOMs, further confirming DLBCL-targeting specificity (Figure 4G). Therefore, SDS-PAGE was used to confirm that final PSOM formulations were not contaminated by nonconjugated Fab, as evidenced by the disappearance of the Fab-DBCO band in the final sample analysis (Figures 4H, S14). Notably, the disappearance of the FAB-DBCO band in αCD19-PSOM samples was due to the intended covalent, rather than artifactual noncovalent, Fab:polymer interaction as spiking additional Fab into the purified PSOM samples restored the presence of the Fab band (Figure 4H). The degree of Fab functionalization, as measured by protein quantification, was found to be 33.4% relative to the theoretical degree of polymer functionalization, assuming a perfect reaction yield between DBCO and N_3_ (i.e., 33.4% of 0.1% of all polymers on the outer PSOM surface was functionalized with Fab; Figure 4I). Flow cytometry was then used to quantify cellular uptake of optimized αCD19-PSOM_calcein_ into DLBCL cells with a range of cell surface CD19 antigen expression. In addition to being time-dependent and dose-dependent, αCD19-PSOM_calcein_ uptake directly correlated with the amount of CD19 expression on various DLBCL cell lines (Figures 4E,F,J and S12, S13). Previous reports have shown related PEG-PPS PSOMs to be nontoxic at high concentrations,^36^ and similarly, brief (24 h) and prolonged (72 h) incubation of αCD19-PSOM_empty_ with DLBCL cells *in vitro* resulted in no evidence of toxicity even at the highest concentration tested, 682 μg/mL (Figure S15).

### αCD19-PSOMs Specifically Target DLBCL *In Vivo*

**Figure 5:**
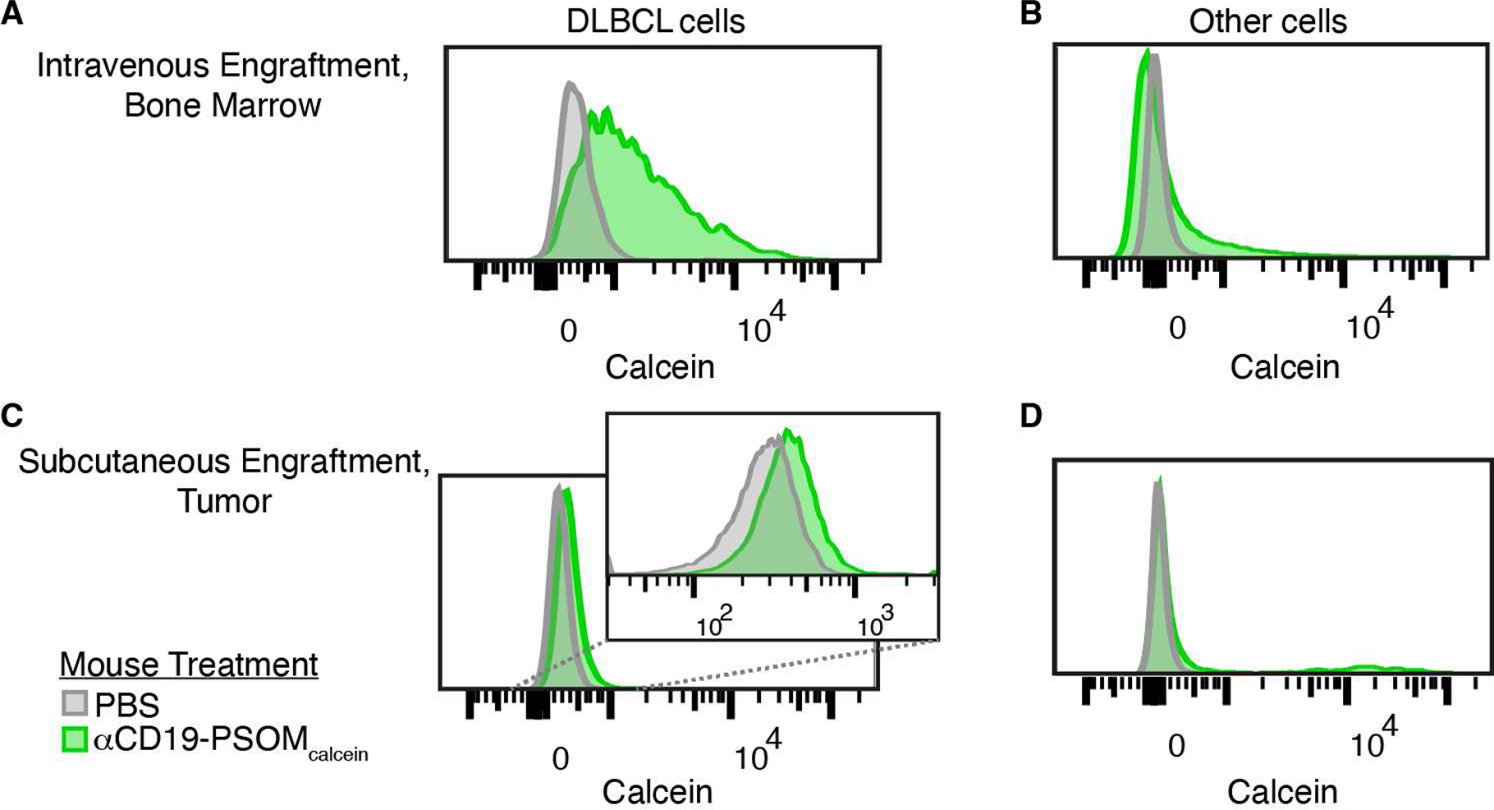
CD19-targeted polymersomes target DLBCL cells *in vivo*. OCI-Ly8 DLBCL cells were engrafted in NSG mice on day 0 either intravenously (A,B) or subcutaneously (C,D). On day 6, the mice were treated intravenously with either PBS (gray) or αCD19-PSOM_calcein_ (green). On day 7, the mice were sacrificed, and single-cell suspensions of bone marrow or a solid tumor were analyzed by flow cytometry. Representative histograms are shown with axis-zoomed inlays where appropriate. (A,B) In the bone marrow of mice with intravenously engrafted DLBCL, (A) αCD19-PSOM_calcein_ was detectable in DLBCL cells (CD19^+^CD20^+^) but (B) not in other cells of the bone marrow. (C,D) In the tumors of mice with subcutaneously engrafted DLBCL, (C) αCD19-PSOM_calcein_ could be detected in DLBCL cells (CD19^+^CD20^+^). (D) Most CD19^–^ cells were unaffected by treatment with αCD19-PSOM_calcein_, except for a small subset of cells, likely macrophages.

The ability of αCD19-PSOM_calcein_ to specifically target CD19^+^ DLBCL *in vivo* was then tested by using xenografted animal models. Mice were engrafted intravenously or subcutaneously with OCI-Ly8 to represent either disseminated or localized (solid) disease, respectively. Six days following engraftment, mice received either a single intravenous injection of vehicle (PBS) or αCD19-PSOM_calcein_, and 24 h later, intracellular calcein uptake was measured using flow cytometry (Figure S16). For mice with disseminated DLBCL, tumor cells were found primarily in the bone marrow and had abundant calcein uptake (Figure 5A), while non-DLBCL cells in the bone marrow had negligible uptake (Figure 5B). Subcutaneously implanted DLBCL showed similarly specific, albeit less, calcein uptake in CD19^+^ cells, while nontumor cells had negligible uptake, similar to the disseminated model (Figure 5C,D). There was a small population of calcein-positive nontumor cells in (D). We hypothesize that these could be macrophages, which are known to be present in NSG mice. These data demonstrated that αCD19-PSOM delivers its cargo specifically to CD19-expressing DLBCL in mouse xenograft models of both disseminated and solid tumor diseases while minimizing delivery into other cell types.

### Polymersome Delivery Maintains ATSP-7041 Efficacy but Improves Tolerability *In Vivo*

**Figure 6:**
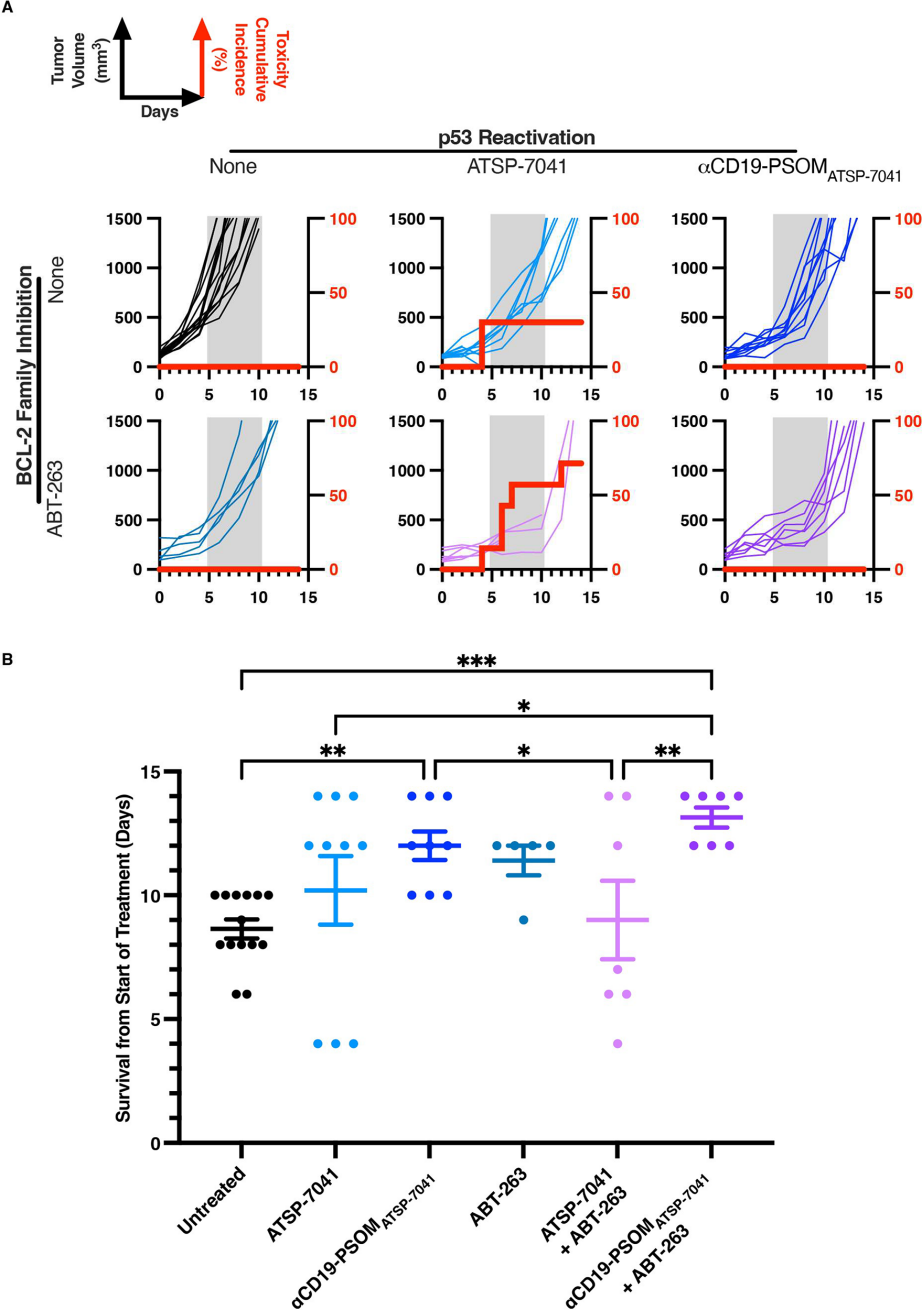
Polymersome delivery maintains ATSP-7041 antitumor efficacy and decreases toxicity *in vivo*. NSG mice (5–14 per group) were engrafted with subcutaneous OCI-Ly19 tumors, and once tumors reached 100 mm^3^, they were treated with nothing (black), ATSP-7041 (30 mg/kg QOD days 1–9; light blue), αCD19-PSOM_ATSP-7041_ (30 mg/kg ATSP-7041 QOD days 1–9; dark blue), ABT-263 (20 mg/kg QD days 1–10; teal), ATSP-7041 (days 1–9) and ABT-263 (days 2–11) (light purple), or αCD19-PSOM_ATSP-7041_ (days 1–9) and ABT-263 (days 2–11) (dark purple). (A) Tumor volumes were measured until the tumor volume reached 1500 mm^3^ or the mice died or became moribund due to drug toxicity. Red lines indicate the cumulative incidence of death due to drug toxicity. The gray shaded region is for visual reference of when untreated mice died due to tumor burden. (B) Survival comparison between the treatment groups. Plotted are the means ± SEM * *p* < 0.05, ** *p* < 0.01, *** *p* < 0.001 using Fisher’s LSD test.

We hypothesized that delivering ATSP-7041 specifically to CD19^+^ DLBCL cells, instead of using no delivery vehicle, would maintain the DLBCL-killing efficacy but decrease toxicity. CD19^+^ (Figure S17) OCI-Ly19 with WTp53 was used as the tumor model.^51^*In vitro*, treatment of OCI-Ly19 cells with free ATSP-7041 or αCD19-PSOM_ATSP-7041_ resulted in indistinguishable DLBCL killing (Figure S18). *In vivo*, OCI-Ly19 grew aggressively as a xenograft; once lesions were measurable (∼100 mm^3^), tumors rapidly expanded such that untreated mice needed to be sacrificed secondary to a large tumor burden (1500 mm^3^) within 6–10 days (Figure 6A; indicated by gray shaded regions). When treated with either intravenous free ATSP-7041 or αCD19-PSOM_ATSP-7041_, the two groups had similar antitumor effects (Figure 6A). However, 30% of mice treated with free ATSP-7041 experienced severe treatment-related toxicity, including death or severe weight loss >20%. Meanwhile, none of the mice in the untreated or αCD19-PSOM_ATSP-7041_ treated groups experienced toxicity. Thus, in this highly aggressive tumor model, the αCD19-PSOM_ATSP-7041_ delivery system significantly delayed tumor growth while minimizing the toxicity of untargeted p53 activation (Figure 6A) and prolonging survival (Figure 6B).

### αCD19-PSOM_ATSP-7041_ Allows for Tolerable Simultaneous p53:BCL-2-Family Therapy in DLBCL *In Vivo*

We next sought to harness the antitumor synergy of dual p53:BCL-2 family therapy while using CD19-targeted polymersome delivery of ATSP-7041 *in vivo*. ABT-263, given daily at 20 mg/kg for 10 days, resulted in delayed tumor growth and no overt toxicity compared to untreated controls (Figure 6A) but no statistically significant increase in survival (Figure 6B). However, the combination of free ATSP-7041 with systemic ABT-263 proved to be lethal in 71% of animals well before completing treatment (Figure 6A). These animals experienced severe weight loss and spontaneous death with macroscopic necrotic organs. In contrast, combining αCD19-PSOM_ATSP-7041_ with systemic ABT-263 resulted in significantly delayed tumor growth and no toxicity (Figure 6A) with a statistically significant increase in survival (Figure 6B).

In summary, p53 reactivation using ATSP-7041 was highly synergistic with BCL-2 family modulation using ABT-263 against DLBCL but also unacceptably toxic *in vivo* (Figure 6A). However, when ATSP-7041 was delivered using CD19-targeted PSOMs (i.e., αCD19-PSOM_ATSP-7041_), the therapeutic synergy was maintained while treatment-related toxicity was eliminated, even in this aggressive DLBCL model (Figure 6A,B).

## Conclusions

New treatment paradigms are needed for patients with chemoresistant cancers, for which systemic treatments with combination therapies represent the only truly realistic clinical options. Here, we present a potential opportunity for therapeutic synergy against tumors by dual direct targeting of the intrinsic apoptotic pathway via reactivating p53 and inhibiting the BCL-2 family of proteins. We found that reactivation of p53 via ATSP-7041 induced significant global proapoptotic transcriptional alterations within the BCL-2 family of proteins, resulting in meaningful apoptotic priming of human DLBCL. ABT-263 exploited this lower threshold for mitochondrial depolarization to induce potent DLBCL killing. While conceptually promising and potent *in vitro*, the combination of HDM2/HDMX-targeting and the BCL-2 family blockade was highly toxic and resulted in a prohibitively narrow therapeutic window *in vivo*. Reactivation of p53 in nontumor tissues has also been a concern for HDM2-targeting small-molecule monotherapies, but toxicity has been observed only when these agents are used at high doses.^28^

Packaging and delivering hydrocarbon stapled peptides in the manner detailed here may offer expanded clinical translation of peptide-based therapeutics and ATSP-7041/ALRN-6924. This strategy could also potentially overcome limitations of the clinical efficacy of small-molecule HDM2 inhibitors, which is thought, at least in part, to result from their lack of affinity for HDMX. Indeed, this lack of HDMX-targeting has been shown to limit the therapeutic efficacy of HDM2-targeting small molecules in tumor cells.^25,52,53^ This may be a particular problem in cases of DLBCL, which have a large array of copy number alterations that decrease p53 activity and where overexpression of *HDM2* and *HDMX* is common.^24,28^

We also present here the packaging and targeted delivery of a hydrocarbon stapled peptide without synthetic modifications. We believe this point may prove critical, as the attachment of terminal residues onto peptides can substantially inhibit the interaction between their therapeutic portion(s) and their protein target binding interface.^54^ The packaging and delivery of unmodified peptides could enhance the efficacy of many preclinically promising stapled peptides, where often the most high-affinity binders lack cellular penetration.^55,56^ A remaining obstacle to more generalized stapled-peptide delivery will be efficient endosomal escape. Unlike ATSP-7041, which can cross lipid membranes (i.e., promoting endosomal escape) readily and without assistance, membrane permeability is a limiting factor for most stapled peptides and requires extensive optimization.^33,57^ On this front, supporting previous similar examples, we have shown that PEG-SS-PPS, with a disulfide bond between the hydrophilic and hydrophobic blocks for endosomal reduction and PSOM destabilization, enhances the intracellular accumulation of model cargo.^37^ However, further research is needed to distinguish between PSOM release with intraendosomal accumulation and endosomal escape with cytoplasmic accumulation, as we have measured for nonstapled peptide amphiphiles.^58^

Beyond contributing to treating DLBCL and packaging unaltered stapled peptides for delivery to cells, the work presented here also highlights the promise of targeted nanomedicines, which offer complementary opportunities to improve the clinical translation of drug candidates. While traditional drugs rely on a single molecule or moiety for their pharmacokinetic and pharmacodynamic properties, nanomedicine platforms like those described here may augment such properties, for molecules that would otherwise fail as therapeutics. Moreover, nanomedicines must not only be efficacious *in vivo* but must also be scalable. In this work, we focused on biocompatible materials and scalable synthesis, assembly, and purification methods with an eye toward eventual clinical translation. PEG-PPS and PEG-SS-PPS nanomaterials have already shown biocompatibility and translational promise in therapeutics, diagnostics, and immune modulation,^36,38−41,59,60^ and this work adds the ability to target PSOMs using the specificity and affinity of F(ab) antibody fragments. A parallel body of research uses CD19-targeted liposomes to deliver chemotherapeutics to DLBCL.^46,61−65^ The CD19-targeted PSOMs presented here aim to expand on this tenet, utilizing the benefits of improved stability, PEGylation, and synthetic flexibility of PSOMs compared to liposomes.^66−68^

In summary, the work demonstrated here exists at the intersection of DLBCL cancer biology, stapled-peptide therapeutics, and targeted nanomedicine. We believe such an antigen-targeted PSOM approach could be adapted to deliver a broad range of payloads (e.g., traditional chemotherapeutics, small-molecule therapeutics, biologic therapeutics, radiosensitizers, and diagnostic markers) through a variety of antigen targets and make meaningful strides against a plethora of cancers.

## Methods/Experimental

### ATSP-7041 Synthesis, Purification, and Characterization

All-hydrocarbon stapled peptides were synthesized on a PreludeX peptide synthesizer (Gyros Protein Technologies) using techniques adapted from those previously described.^69,70^ Briefly, rink amide AM low-loading resin (Sigma-Aldrich) was used, and deprotection reactions were performed with 20% piperidine in NMP for 2 × 10 min, except for stapling amino acids, which were deprotected for 4 × 10 min. Unless otherwise specified, coupling reactions used 10 equiv of amino acid (Gyros Protein Technologies; 300 mM solution in NMP), 9.5 equiv of HATU (285 mM solution in NMP), and 20 equiv of DIPEA (600 mM solution in NMP) for 30 min. Stapling amino acids (Sigma-Aldrich; Advanced ChemTech) were coupled using half that amount of each solution for 1 h. To couple the amino acid directly following a stapling amino acid, the coupling reaction was repeated for 4 × 1 h, except Cba, which was repeated for 2 × 4 h. After each coupling reaction, the resin was exposed to capping solution (4/1/0.1 NMP/Ac_2_O/DIPEA) for 10 min to cap any unreacted amines and generate truncation impurities instead of deletion impurities to simplify HPLC purification. After each reaction step, the resin was washed with alternating washes of DMF and DCM. After the linear synthesis was complete, the N-terminus was deprotected and acetylated with capping solution. For RCM stapling, the resin was washed thoroughly with DCM and then suspended in a 4 mg/mL solution (prepared fresh at the start of synthesis) of Grubbs first generation catalyst in anhydrous 1,2-dichloroethane with 20 mol % catalyst with respect to resin substitution. Stapling reactions were carried out under nitrogen bubbling for 3 × 2 h, then 3 × 4 h with DCM washing between cycles, and confirmed by LCMS through the loss of ethylene (−28 Da). The resin was washed with DCM, dried, cleaved with fresh 95/2.5/2.5 TFA/H_2_O/TIS solution for 2 h, and washed with additional solution. The peptide was precipitated using 50/50 hexane/diethyl ether in 50 mL centrifuge tubes at a volume ratio of 10:1 or greater. The solution was chilled at −80 °C for 1 h, and then, the peptide was pelleted by centrifugation at 1500*g* for 20 min at −10 °C, dried, resuspended in a H_2_O/ACN mixture, and lyophilized. Complete deprotection of the carbamic acid on tryptophan side chains, as identified by MW + 44 Da impurities in LCMS, was facilitated by overnight incubation in 50/50 H_2_O/ACN with ammonium bicarbonate buffer at neutral pH.^69^ When the peptide occasionally precipitated, a large quantity of urea was dissolved into the solution and sonicated. Peptide solutions were filtered and then purified via reverse-phase HPLC-MS using a C18 column (Waters XBridge Peptide BEH C18, 130 Å, 5 μm, 19 × 150 mm) with mobile phases A (water + 0.1% formic acid) and B (ACN). Pure fractions were pooled and lyophilized, redissolved in 30% ACN in H_2_O, filtered, aliquoted, lyophilized, confirmed pure by LCMS, and quantified by amino acid analysis (AAA; UC Davis Molecular Structure Facility).

LCMS was used to confirm the completion of stapling reactions, measure peptide purity, and measure peptide concentrations in polymersome formulations. An analytical column (Waters XBridge Peptide BEH C18, 130 Å, 5 μm, 4 × 150 mm) was used with mobile phases A (water + 0.1% TFA) and B (ACN). Peptide purity was calculated by integrating the 220 nm absorbance chromatogram and was always >95%. ATSP-7041 was eluted as two isomers (Figure S4), as previously shown.^71^ Peptide concentration in polymersomes was compared to an AAA-quantified standard sample using the area under the curve of the peptide peak’s UV absorbance. PEG-SS-PPS polymer interacted strongly with the column, which required occasional washing with acetonitrile, DCM, and then acetonitrile.

### Fab Design, Production, and DBCO Functionalization

#### Fab Design

The αCD19 Fab was designed using published variable region sequences (V_k_ and V_H_) from HD37 mouse-antihuman-CD19 IgG for both light chain (GenBank CAA67620, amino acids 1–111) and heavy chain (GenBank CAA67618, amino acids 1–124),^72,73^ combined with constant regions (C_k_ and C_H_) from mouse IgG consensus sequences for light chain (UniProt P01837, amino acids 1–107) and heavy chain (UniProt P01868, amino acids 1–104). For a control Fab, the variable regions were substituted for those from an antibody specific for OspA without changing the constant regions.^74,75^ A cysteine linker (···GSGGSSGSGC) was encoded on the C-terminus of the heavy chain to create αCD19-cys and αOspA-cys for site-specific conjugation to polymersomes. Sequences can be found in Figure S9.

#### Fab Cloning

Fab sequences were acquired as gBlocks Gene Fragments (Integrated DNA Technologies) and cloned into an AbVec2.0 plasmid under cytomegalovirus (CMV) promoter for constitutive mammalian expression.^76^ A signal peptide sequence derived from osteonectin was added to the N-termini of both light and heavy chains to induce protein secretion. After cloning and transformation into competent DH5α, the plasmid was selected for using ampicillin and isolated using NuelcoBond Xtra Maxi kits (Machery Nagel). Purified plasmids were sequenced by using the University of Chicago Comprehensive Cancer Center DNA Sequencing and Genotyping Facility (UCCCC-DSF).

#### Fab Expression, Purification, and Quantification

Fabs were expressed in HEK293T suspension cells in a FreeStyle 293 Expression Medium (Thermo Fisher Scientific). At 1 million cells/mL in log-phase growth, cells were transfected with 1 μg of plasmid and 2 μg of polyethylenimine in 40 μL of OptiPRO SFM (Gibco) per million cells. Transfected cells were cultured for 6 days in shake flasks at 37 °C and 5% CO_2_. The cells were then pelleted by centrifugation, and the supernatant was filtered through a 0.22 μm filter and pH-adjusted to 7.0 using 1 M Tris buffer, pH 9.0. The Fabs were purified by affinity chromatography using 5 mL of HiTrap Protein G HP columns (GE Life Sciences) via fast protein liquid chromatography (AKTA FPLC, GE Healthcare). A dedicated column was used for each Fab sequence to prevent cross-contamination. Up to 3 × 5 mL columns were connected in series for large-scale purification. The column was first equilibrated with 5 column volumes (CVs) of PBS at 5 mL/min. The crude Fab solution flowed over the column at 5 mL/min, and the column was washed with 10 CVs of PBS. Pure Fab was eluted with 0.1 M glycine-HCl, pH 2.7, into 3 mL fractions prebuffered with 125 μL each of 1 M Tris buffer, pH 9.0, and 1 mL of 1× PBS, pH 7.4, to achieve neutral pH in each fraction. The crude flow-through was collected, and the purification was repeated multiple times until the UV absorbance of the elution peak was minimal. Elution peaks were pooled, dialyzed extensively (Slide-A-Lyzer, G2 Dialysis Cassettes, 10 kDa MWCO, Thermo Fisher Scientific) against 1× PBS, pH 7.4, concentrated (Amicon Ultra-15, 10 kDa MWCO, Millipore Sigma) to no more than 10 mg/mL, sterile-filtered, and either stored at 4 °C or aliquoted and frozen for later use. Fab concentrations were calculated using UV absorbance based on their calculated extinction coefficients at 280 nm (48 923 M^–1^cm^–1^ for αCD19-cys and 47 432 M^–1^cm^–1^ for αOspA-cys).

#### Fab Functionalization with DBCO

Coomassie-stained SDS-PAGE was used to determine the percentage of each sample that was unimeric, intact Fab (>80%), as opposed to Fab-Fab disulfides or free heavy/light chain, which were the two other minor bands in some samples (e.g., Figure S10B). The concentration of unimeric, intact Fab was calculated as the product of the concentration determined by UV absorbance at 280 nm and the percentage determined by SDS-PAGE. EDTA (UltraPure, 0.5 M EDTA, pH 8.0; Invitrogen) was added to a final concentration of 10 mM to the Fabs in PBS, at pH 7.4. TCEP, aliquoted in Milli-Q water and frozen at 1 M, was diluted immediately before use to 1 mM in PBS with 10 mM EDTA, pH 7.4. TCEP (0.85 equiv with respect to the concentration of intact, unimeric Fab) was added to the Fab, and the reaction was immediately vortexed. The reaction was incubated at 37 °C for 90 min. The heterobifunctional linker (sulfo DBCO-PEG4-maleimide; Click Chemistry Tools) was dissolved immediately before use at 20 mM in PBS with 10 mM EDTA, pH 7.4. 100 equiv of linker was added to the reduced Fab without workup, and the reaction was immediately vortexed and incubated at room temperature for 1 h. After 1 h, the Fab was immediately purified by eight rounds of diafiltration into 1× PBS, pH 7.4, at 4 °C, using Amicon ultrafiltration devices with a 10 kDa MWCO and a volume appropriate to the scale of the reaction to avoid concentrating the Fabs to greater than 10 mg/mL. Functionalized Fabs were then sterile-filtered. The Fab concentration was then calculated using the equation:
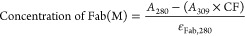
1with *A*_280_ and *A*_309_ are the sample absorbance at 280 and 309 nm, respectively, the correction factor , and ε_Fab,280_ the calculated extinction coefficient of the Fab at 280 nm. DBCO concentration was calculated using the equation:
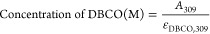
2with ε_DBCO,309_ = 12 000 M^–1^ cm^–1^. The number of DBCO groups per Fab was calculated as the ratio of their concentrations. DBCO-functionalized Fabs were stored at 4 °C if they were to be used within a month, and the rest were aliquoted and frozen for storage until use.

### Polymersome Synthesis

#### Synthesis of Poly(propylene sulfide) (PPS) with Pyridyl Disulfide (PDS) End-Group (PPS-PDS)

PPS was synthesized by living anionic ring-opening polymerization (Figure S5A) with the following adaptations to previously reported methods.^37^ Benzyl mercaptan (1 equiv) in degassed, anhydrous THF (20 mM) was deprotonated with sodium methoxide (NaOMe; 1.1 equiv) under nitrogen protection for 30 min. Propylene sulfide (53.3 equiv) was rapidly added by syringe under vigorous stirring and nitrogen protection. The reaction was carried out under a constant flow of vented nitrogen protection to prevent pressure accumulation. The reaction proceeded to completion within 1 h, according to ^1^H NMR, at which point the thiolates were quenched with acetic acid (AcOH; 2 equiv). Disulfide-dimerized PPS chains were reduced by adding triethylamine (TEA; 3 equiv), water (H_2_O; 8 equiv), and tributylphosphine (TBP; 8 equiv) under nitrogen protection for 4 h. TBP can spontaneously ignite upon contact with oxygen and thus was handled under inert gas. Aldrithiol-2 (25 equiv) was dissolved in a minimal amount of THF and degassed, and the PPS reaction mixture was cannulated dropwise into the capping solution under nitrogen protection and vigorous stirring and stirred overnight. THF was then evaporated, and the yellow crude oil was extracted with methanol repeatedly until colorless. Removal of aldrithiol-2 and the mercaptopyridine byproduct were confirmed by silica TLC with a mobile phase of 2% methanol in DCM. The fluorescence indicator under UV light was used to detect aldrithiol-2 and mercaptopyridine. CAM staining was used to detect PPS-PDS. Dragendorff staining was used to detect mercaptopyridine. Pure PPS-PDS was dried under a high vacuum. Purity was confirmed by DMF GPC (Figure S5B), NMR (Figure S5C), and TLC. PPS-PDS was stored under argon protection at −80 °C. This synthesis was successfully scaled up to 375 g scale with 99.8% yield using a 6 L flat-bottom flask and laboratory-scale Schlenk line.

#### Synthesis of Methoxy- and Azide-Poly(ethylene glycol)-*block*-poly(propylene sulfide) (mPEG-SS-PPS and N_3_-PEG-SS-PPS)

Thiol-functionalized PEG polymers were purchased from Laysan Bio Inc. (mPEG-SH) and Nanosoft Polymers (N_3_-PEG-SH) and used as delivered. Molecular weights of PEGs were measured by NMR and MALDI to be approximately 1200 Da, and our PPS degree of polymerization (DP) was scaled accordingly to maintain previously reported block ratios. PPS-PDS (1.2 equiv PDS) and R-PEG-SH (R = OMe or N_3_; 1 equiv free thiol (as determined by polymer mass and dimerization degree by GPC)) were each dissolved in DCM (1 and 0.01 g/mL respectively) and degassed under nitrogen bubbling. The PEG solution was cannulated dropwise into the PPS solution under vigorous stirring and allowed to react overnight. The crude product was concentrated and purified over a gradient silica flash column. Briefly, 30 g of dry silica per gram of crude mixture (assuming no solvent) were loaded into a flash column as a slurry in DCM. The concentrated sample was loaded onto the column in DCM, in which there was minimal migration. The column was then washed with 2% methanol in DCM, in which PPS-PDS and PPS-PPS disulfides were washed off the column. Due to the refractive index matching of the silica and solvent, this migration was visible by eye as an opaque band. The yellow mercaptopyridine byproduct was also visibly eluted in this washing step. The PEG-SS-PPS band, still visible at the top of the column, was then eluted with 10% methanol in DCM. Behind the eluting band, the silica visibly turned opaque as the methanol saturated the silica. The solvent from the eluted product was then removed by rotary evaporation. A minimal amount of DMF was used to transfer the polymer to 50 mL centrifuge tubes. The polymer was precipitated with −20 °C MeOH at a volume ratio of 1:10 or greater and centrifuged at 4700*g* at −10 °C until the supernatant was visibly clear. The deceleration rate was minimized to avoid disturbing the oil when the centrifuge stopped. The supernatant was decanted, and the oil was then extracted twice more with −20 °C MeOH, centrifugation, and decanting. After the MeOH extractions, the removal of DMF and coeluting PEG was confirmed by NMR and TLC with CAM staining and a mobile phase of 8% methanol in DCM. The polymer was redissolved in DCM, filtered through a 0.2 μm filter into preweighed scintillation vials, and dried by rotary evaporation followed by a high vacuum. The final product was confirmed to be pure by DMF GPC (Figures S6A and S7A), NMR (Figures S6B and S7B), and TLC. All polymers were stored under argon protection at −80 °C. This synthesis was successfully scaled up to a 10 g scale of purified mPEG-SS-PPS using laboratory-scale equipment with a representative yield of 12%, presumably due to disulfide shuffling in the reaction.

#### Thin-Film Polymersome Assembly

Polymers were dissolved in DCM, and 10 mg was transferred to a 2 mL piranha-etched glass vial. The DCM was evaporated under a high vacuum to form a thin layer of polymer film on the glass walls. Next, 250 μL of sterile PBS was added to the vial, and the vial was slowly rotated at room temperature for 2–3 days until no polymer was visible on the vial walls.

#### Flash Nanoprecipitation (FNP) Polymersome Assembly

We 3D-printed a CIJ-D device (Figure 3B,C) using the same design parameters previously reported^77^ and used by others to assemble PEG-PPS polymersomes.^35,36^ Syringe adapters (IDEX P604) and outlet adapters (IDEX P202X and IDEX P200X) were purchased from Fisher Scientific. The outlet tubing used was 1/16″ OD and 0.04″ ID. Before each use, the device was sterilized and cleaned with 0.5 M NaOH and repeatedly rinsed with Milli-Q water. All assemblies were done in a sterile hood, following the protocols and ratios previously described.^35,36^

#### Calcein Polymersome (PSOM_calcein_) Encapsulation

A 100 mM calcein solution was prepared at 313 mOsm. Calcein in its protonated form (Calcein High Purity, Thermo Fisher Scientific) was dissolved in 2 mol equiv of NaOH from a 1 M solution in water, and then, 1× PBS, pH 7.4 (Gibco, Thermo Fisher Scientific), was added to contribute 13 mOsm. The solution was diluted to a final calcein concentration of 100 mM using Milli-Q water to achieve a final osmolarity of 313 mOsm. This solution was used as the antisolvent stream and the dilution reservoir during FNP encapsulation.

#### ATSP-7041 Polymersome (PSOM_ATSP-7041_) Encapsulation

A peptide:polymer mass ratio of 1:4 was used. The polymer was dissolved in THF at 40–100 mg/mL with 95% mPEG-SS-PPS and 5% N_3_-PEG-SS-PPS. Lyophilized ATSP-7041 was dissolved in DMSO at 50 mM and added to the polymer THF solution. For FNP, the THF solution was impinged against an equivalent volume of PBS into a PBS reservoir five times the volume of the THF solution.

#### Polymersome Extrusion

All polymersome samples were extruded 11–21 times through a 100 nm pore-size membrane (Whatman Nucleopore Track-Etched Membrane, 19 mm, 100 nm) using a syringe-driven Mini Extruder (Avanti Polar Lipids) in a sterile hood. Size and dispersity were monitored by DLS. DLS measurements were repeated and averaged until the correlation function reliably fit the data. The polymersomes were then immediately purified from residual organic solvents using gravity-driven disposable PD-10 desalting columns containing Sephadex G-25 resin (GE Healthcare) into 1× PBS, pH 7.4 (Gibco, Thermo Fisher Scientific).

#### Fab Conjugation to Polymersomes

Polymersomes were assembled with 5% N_3_-PEG-SS-PPS and 95% mPEG-SS-PPS. DBCO-functionalized Fabs were then reacted with N_3_-functionalized polymersomes with Fab-DBCO as the limiting functional group. The click reaction was allowed to proceed overnight at room temperature. The samples were then either purified or transferred to 4 °C until purification. The “theoretical Fab density” (Figures 4E, S12) was calculated as a percentage of the polymers in the outer layer of the polymersome bilayer (half of the total polymer), assuming 100% N_3_:DBCO yield. The yield was measured for a representative purified sample using CBQCA protein quantification (Molecular Probes; Figure 4I).

#### Fab-Polymersome Purification

Fab-functionalized polymersomes were purified by size into PBS either by gravity-driven SEC using Sepharose CL-4B resin or by diafiltration using TFF (MicroKros, 300 kDa MWCO, mPES, 0.5 mm; Repligen) driven either by syringe or, at larger scales, by a peristaltic pump (Figure 3E). The gravity column or TFF flow path was first sterilized by using 0.5 M NaOH and then equilibrated with PBS before purification, all in a sterile hood. For TFF driven by a peristaltic pump, the pump (Fisher Scientific, 13–876–2) was set up with tubing on the pump spindle (3/32″) such that a medium speed (i.e., 40–50) corresponded to no more than 12 mL/min. The sample was drawn through the setup shown in Figure 3E. A slight amount of backpressure, only enough to slightly slow the flow rate, was generated to increase the filtration rate by using a screw compressor clamp (Humboldt H-8665) on the outlet tubing from the MicroKros outlet to the sample reservoir. This setup’s dead volume was approximately 2 mL.

#### Fab-Polymersome Characterization

For purified formulations, the peptide concentrations were measured by LCMS against an AAA-quantified sample. Polymer concentrations were measured by GPC against a standard sample of known concentration using refractive index AUC. Fab conjugation was confirmed with SDS-PAGE gel (e.g., Figures 4H, S14). All gel samples were prepared with sodium azide (to quench DBCO:azide reactions) and NEM (to quench thiols and disulfide shuffling). Broad polymer smearing occurred in the presence of sodium azide in the gel samples. Fab concentrations were measured using CBQCA against a UV–vis quantified Fab-DBCO control (e.g., Figure 4I).

### Drug Treatments

#### *In Vitro* ATSP-7041 and ABT-263 Treatments

Lyophilized ATSP-7041 or ABT-263 was dissolved in DMSO at 20 mM and then diluted into cell culture medium for cell treatments. DMSO solutions and lyophilized powders were stored at −80 °C when not in use.

#### *In Vivo* ATSP-7041 Treatment

ATSP-7041, redissolved in DMSO less than 2% of the final formulation volume, was solubilized by mixing with DSPE-PEG(2000) (powder, Avanti Polar Lipids) at a 3:50 mass ratio and PBS pH 7.4 to a final peptide concentration of 3 mg/mL followed by sonication at 50 °C for at least 20 min until transparent. This solution was then passed through an 800 nm extrusion membrane (Whatman Nucleopore Track-Etched Membrane, 19 mm, 800 nm; Avanti Polar Lipids Mini Extruder) 11 times to extrude any aggregates, and the solution was kept sterile and stored at 4 °C until use. This procedure was adapted based on similar DSPE-PEG solubilization protocols described in the literature.^25,26^ For this study, all formulations were prepared shortly before use, although DSPE-PEG has been shown to be stable to hydrolysis during long-term storage.^78^ ATSP-7041 solution, or αCD19-PSOM_ATSP-7041_, was injected by tail vein at doses of 30 mg peptide per kg mouse weight every other day, as previously published,^25−27^ for five doses.

#### *In Vivo* ABT-263 Treatment

ABT-263 (AbbVie) was formulated in 60% Phosal 50 PG, 30% PEG 400, and 10% EtOH as previously described.^12^ The final concentration of ABT-263 in the solution was 2 mg/mL for 20 mg/kg dosing and 10 mg/mL for 100 mg/kg dosing. The solution was stored at room temperature, protected from light, and made fresh every 5–7 days. Mice were treated by oral gavage daily for 10 days.

### Xenograft Experiments

NOD.Cg-Prkdc^scid^ Il2rg^tm1Wjl^/SzJ (NSG) mice (Jackson Laboratory) were housed at the University of Chicago Animal Resource Center. Experiments were conducted under the guidelines and regulations of the Institutional Animal Care and Use Committee of the University of Chicago.

For subcutaneous xenografts, the engrafted OCI-Ly19 or OCI-Ly8 cells were suspended in either PBS or 50% matrigel in PBS with 5 million cells in no more than 200 μL per injection and engrafted on the left hind flank. Treatments began the day after tumor volume reached 100 mm^3^ when mice were randomly assigned to a treatment group. Subcutaneous tumor volume was calculated as . Tumor volume and mouse weight were measured every other day until reaching a euthanasia end point: tumor volume 1500 mm^3^, 20% weight loss, or when the animal became moribund.

For disseminated xenografts, 5 million cells in 200 μL of PBS per injection were injected into the tail vein. Mouse weights were measured 2–3 times per week until reaching a euthanasia end point: 20% weight loss or when the animal became moribund. Luciferase-expressing OCI-Ly19 (OCI-Ly19-Luc) tumor burden was measured using bioluminescence imaging with a Xenogen IVIS Spectrum (Caliper Life Sciences) after injection of 150 mg/kg of d-luciferin (Promega). 21 days after inoculation, mice were divided into groups with equivalent disease burden, as determined by bioluminescence imaging, and treatment began the following day. Disease burden was monitored by serial bioluminescence imaging and quantified using the Living Images software package (Caliper Life Sciences).

### Cell Culture

Human DLBCL cell lines were maintained in RPMI 1640 (Gibco, Thermo Fisher Scientific) supplemented with 10% FBS, 10 mM HEPES (Gibco, 1 M), 2 mM l-glutamine (Gibco, 200 mM), 1× MEM nonessential amino acids (Gibco, from 100× solution), and 100 U/mL penicillin-streptomycin (Gibco, 10 000 U/mL) at 37 °C and 5% CO_2_. SU-DHL-5 was purchased from ATCC. OCI-Ly3 and OCI-Ly19 were purchased from DSMZ. OCI-Ly1, OCI-Ly8, and DOHH-2 were kindly provided by the Kline laboratory (University of Chicago). Most cells were split every 2–3 days to 0.5 million cells per mL, but SU-DHL-5 and OCI-Ly3 were split to 0.1 million cells per mL or lower and not allowed to reach densities higher than 1 million cells per mL due to their intolerance to higher densities.

### Quantitative Real-Time PCR (qRT-PCR)

Following appropriate drug treatment (24 h; 1 μM ATSP-7041 or an equivalent volume of DMSO vehicle), cells were lysed with Trizol (Life Technologies), and total RNA was isolated from each sample using the Direct-zol RNA MiniPrep kit (Zymo Research) per the manufacturer’s instructions and quantified (DeNovix DS-11 Spectrophotometer). RNA from each biological replicate (500 ng) was converted to double-stranded cDNA using the Superscript III first strand synthesis reverse transcription kit (Invitrogen) per the manufacturer’s instructions.

qRT-PCR was performed using a TaqMan Master Mix and Gene Expression Probes (Applied Biosystems) for each of the following genes: A1: Hs00187845, B2M: Hs00984230, BAD: Hs00188930, BAK: Hs00832876, BAX: Hs00180269, BCL2: Hs00608023, BCLW: Hs00187848, BCLXL: Hs00236329, BID: Hs00609632, BIM: Hs00708019, BMF: Hs00372937, CDKN1A: Hs00355782, GAPDH: Hs02758991, MCL1: H01050896, NOXA: Hs00560402, PUMA: Hs00248075. Samples were run on the 7500 Fast Real-Time PCR System (Applied Biosciences). Data were analyzed with ExpressionSuite software, utilizing the ΔΔCT method with GAPDH and B2M as two housekeeping genes and DMSO-treated cells as reference samples.

### BH3 Priming

Cells were first treated for 24 h with 2 μM ATSP-7041 or an equivalent volume of DMSO vehicle. BH3 priming experiments were performed as previously described.^79^ Fluorescence was measured at 90 min and normalized to DMSO and FCCP treatment. Data were collected in triplicate.

### Cell Death Assays

Treatments were prepared in 96-well plates in 50 μL per well at 2× treatment concentration and mixed with 10 000 cells in 50 μL. The plates were incubated for 24–72 h, as indicated in each experiment, and then, 100 μL of CellTiter-Glo 2.0 (Promega) was added and pipet-mixed, followed immediately by luminescence reading (SpectraMax iD5, Molecular Devices). ABT-263 sensitivity after ATSP-7041 priming was measured by pretreating cells for 24 h with ATSP-7041 (5 μM for OCI-Ly3, 2 μM for all other cell lines) or an equivalent volume of DMSO vehicle, washing, treating with dose titrations of ABT-263 for 24 h, and measuring viability as above.

### Flow Cytometry

Mouse Fc block (TruStain FcX (antimouse CD16/32) antibody, 101320), APC antihuman CD19 (363006, clone SJ25C1), and APC-Cy7 antihuman CD20 (302314, clone 2H7) were purchased from BioLegend. Human Fc block (BD Biosciences 564220, clone 3070) was purchased from Fisher Scientific. Live/dead (L/D) staining was performed with either an Invitrogen Fixable Blue Dead Cell Stain (L23105) or Zombie Violet Fixable Viability Kit (BioLegend 423113). Anti-Fab F(ab′)_2_ (Alexa Fluor 647 AffiniPure F(ab′)_2_ Fragment Donkey Anti-Mouse IgG (H+L); Jackson ImmunoResearch; 715–606–151) was used to detect the murine-backbone Fabs. Flow cytometry was performed following washing cells in PBS and staining with L/D stain 1:500 in PBS for 15 min on ice. Fc block was added directly to the mixture (1:200 for human Fc block, 1:50 for mouse Fc block) for 15 min on ice. Antibodies were then added (final dilution 1:100) for 30 min on ice. Cells were centrifuged, resuspended in a FACS buffer (5% FBS in PBS), and then analyzed.

### Calcein Polymersome Uptake Measurements

A self-quenching calcein solution was encapsulated in PEG-SS-PPS polymersomes with 5% N_3_ functionalization. Aliquots of this stock solution were then functionalized with either αCD19 or nontargeted (αOspA) Fabs at various Fab:polymer densities. Cells were treated as indicated and analyzed by flow cytometry and ImageStream imaging cytometry. Calcein concentrations were held constant across samples using calcein absorbance after Triton X-100 disruption and calcein dequenching.

### Measuring Polymersome Stability in Serum via Calcein Fluorescence Dequenching

Polymersomes encapsulating a self-quenching calcein solution were assembled, as described above. The resulting stock solution was diluted 1:100 into either RPMI 1640 (“media”), media +10% fetal bovine serum (FBS), or media + 10% FBS + 5 mM Triton X-100 in a black, flat-bottomed 96-well plate. Samples were incubated at 37 °C, and the calcein fluorescence was monitored for 1 h via plate reader (SpectraMax iD5, Molecular Devices). Each sample was prepared in quadruplicate, and each value was background-subtracted using corresponding samples prepared by adding a PBS vehicle instead of polymersomes into the indicated solution, though all background solutions had negligible fluorescence values.

## Supporting Information Available

Figure S1: DLBCL cell death sensitivity to ATSP-7041. Figure S2: Changes in mRNA expression of the BCL-2 family in response to p53 reactivation by ATSP-7041. Figure S3: Cell death sensitivity to ABT-263 after priming with ATSP-7041. Figure S4: LCMS analysis of purified ATSP-7041. Figure S5: Characterization of PPS-PDS. Figure S6: Characterization of mPEG-SS-PPS. Figure S7: Characterization of N3-PEG-SS-PPS. Figure S8: Characterization of PSOM_ATSP-7041_. Figure S9: DNA coding sequences of αCD19 and αOspA Fabs and their protein translations. Figure S10: Expression and binding validation of αCD19 and αOspA Fabs. Figure S11: Full Coomassie-stained SDS-PAGE gels from Figure 4C. Figure S12: CD19-targeting enhances polymersome delivery of calcein into various DLBCL cell lines. Figure S13: Polymersome uptake into DLBCL cell lines depends upon CD19 cell surface expression and polymersome dose. Figure S14: Full Coomassie-stained SDS-PAGE gel from Figure 4H. Figure S15: PEG-SS-PPS is nontoxic at high concentrations. Figure S16: Representative gating strategy for flow cytometry of DLBCL cells from xenografts. Figure S17: OCI-Ly19 expresses CD19. Figure S18: Polymersome delivery maintains the efficacy of ATSP-7041 (PDF)
File S1: Computer-aided design (CAD) file of the 3D-printed flash nanoprecipitation (FNP) device used in this work (ZIP)
