# Supplementary material for: Targeted Polymersome Delivery of a Stapled Peptide for Drugging the Tumor Protein p53:BCL-2-Family Axis in Diffuse Large B-Cell Lymphoma
Source: ACS Nano. 2023 Sep 9;17(23):23374–90. doi: 10.1021/acsnano.3c04112 (PMC10722602; doi:10.1021/acsnano.3c04112)
Supplement: Supplementary file 1 — nn3c04112_si_001.pdf [file nn3c04112_si_001.pdf]

Supporting Information For:

Targeted polymersome delivery of a stapled peptide for drugging the tumor protein p53:BCL-2-family axis in diffuse large B-cell lymphoma

Mathew R. Schnorenberg[1,2,3], Katrina M. Hawley[2], Anika T. Thomas-Toth[2], Elyse A. Watkins[1], Yu Tian[1,2], Jeffrey M. Ting[1], Logan B. Leak[2], Isadora M. Kucera[1,2], Michal M. Raczy[1], Andrew L. Kung[4], Jeffrey A. Hubbell[1], Matthew V. Tirrell[1,], James L. LaBelle[2,*]*

[1]Pritzker School of Molecular Engineering, University of Chicago, Chicago, IL 60637, USA.
[2]Department of Pediatrics, Section of Hematology/Oncology, University of Chicago, Chicago, IL 60637, USA.
[3]Medical Scientist Training Program, Pritzker School of Medicine, University of Chicago, Chicago, IL 60637, USA.
[4]Department of Pediatrics, Memorial Sloan Kettering Cancer Center, New York, NY 10065, USA.

*Corresponding Authors:

James L. LaBelle
900 East 57th Street, KCBD 5122
Chicago, IL 60637
Email: jlabelle@peds.bsd.uchicago.edu
Phone: 773-702-6812
Fax: 773-834-1329

Matthew V. Tirrell
Pritzker School of Molecular Engineering
5640 South Ellis Avenue
Chicago, IL 60637
Email: mtirrell@uchicago.edu
Phone: 773-834-2001
Fax: 773-834-7756

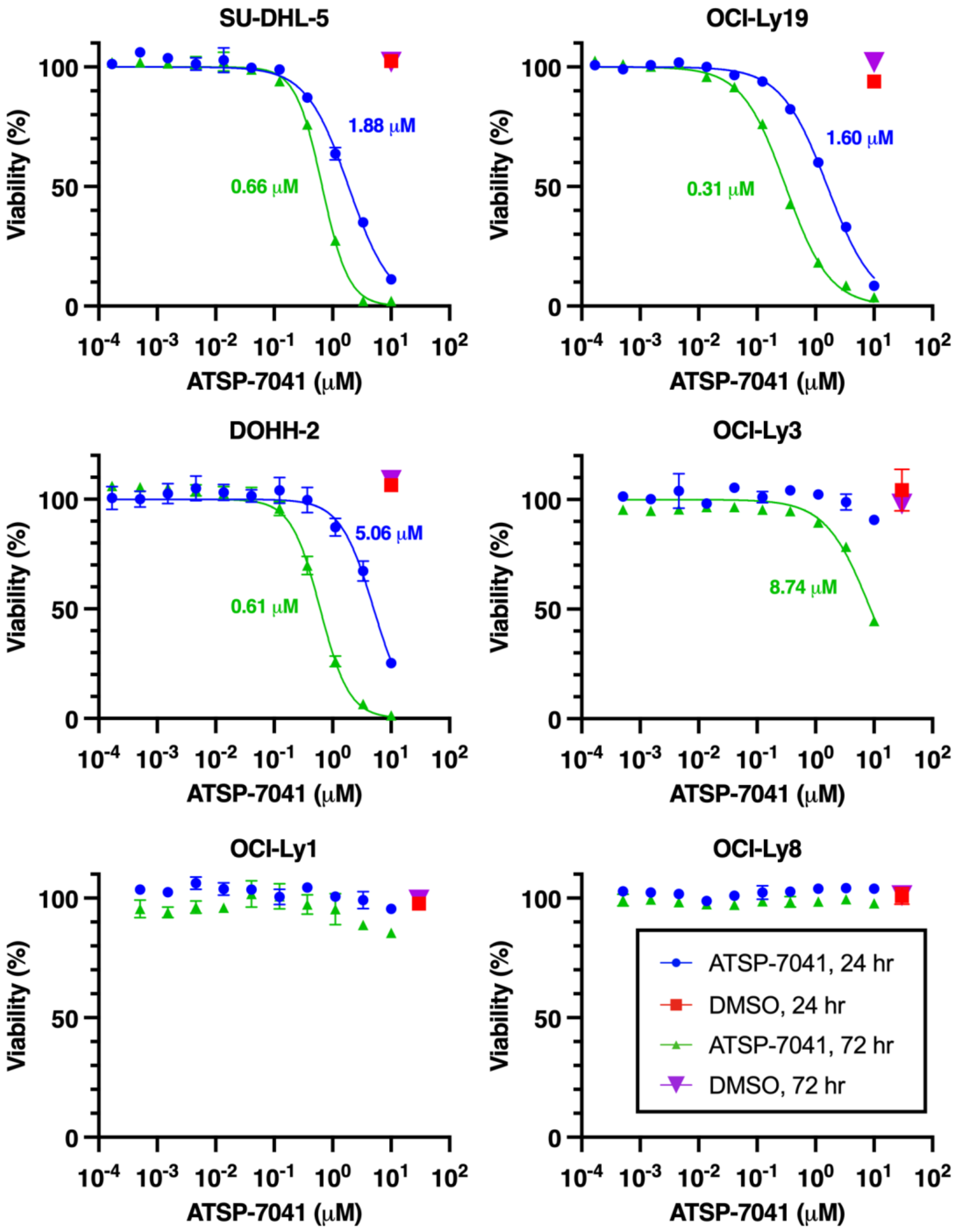

Figure S1: DLBCL cell death sensitivity to ATSP-7041. DLBCL cell lines were treated with ATSP-7041 or DMSO vehicle for 24 or 72 hr, and viability was measured. Data were normalized to untreated cells from the same time point, plotted as the mean of duplicates ± SEM, and fitted using nonlinear regression. EC_{50} is shown. Any absent error bars are smaller than the plotted point.

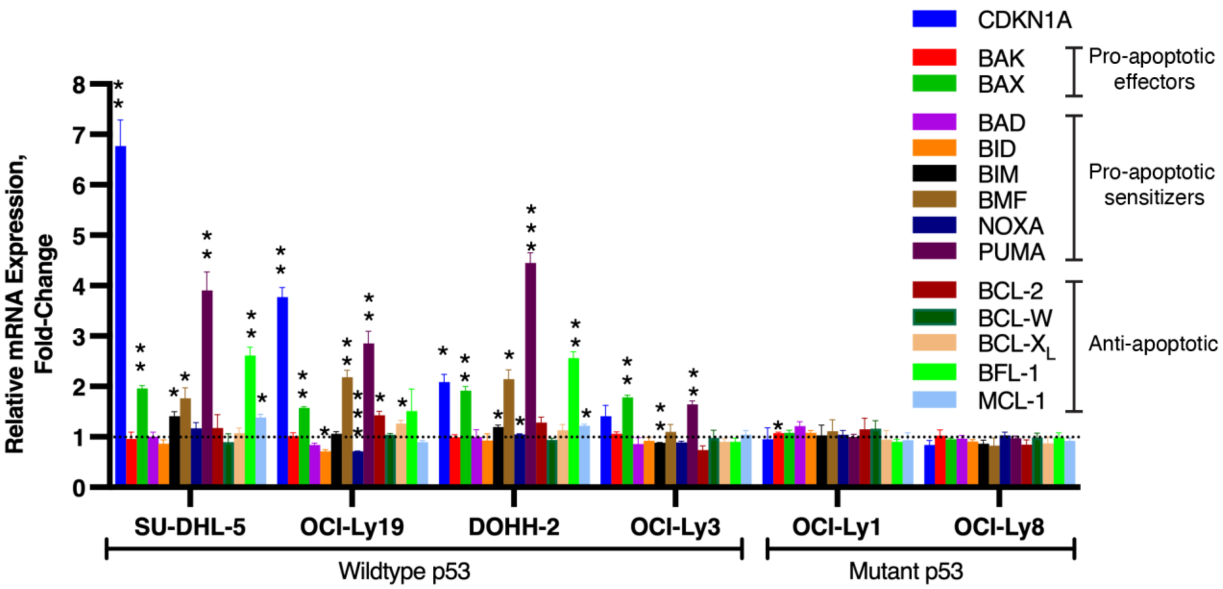

Figure S2: Changes in mRNA expression of the BCL-2 family in response to p53-reactivation by ATSP-7041. DLBCL cell lines were treated with 1 μM ATSP-7041 for 24 hr, and mRNA changes in the BCL-2 family were measured by qRT-PCR. Plotted values are the mean of the relative fold change of biological triplicates ± SEM, using GAPDH and B2M as housekeeping genes and DMSO-treated samples as reference controls. Changes were deemed significant via one-sample t-test (H_0: ΔΔCT = 0; * $p < 0.05$, ** $p < 0.01$, *** $p < 0.001$, **** $p < 0.0001$).

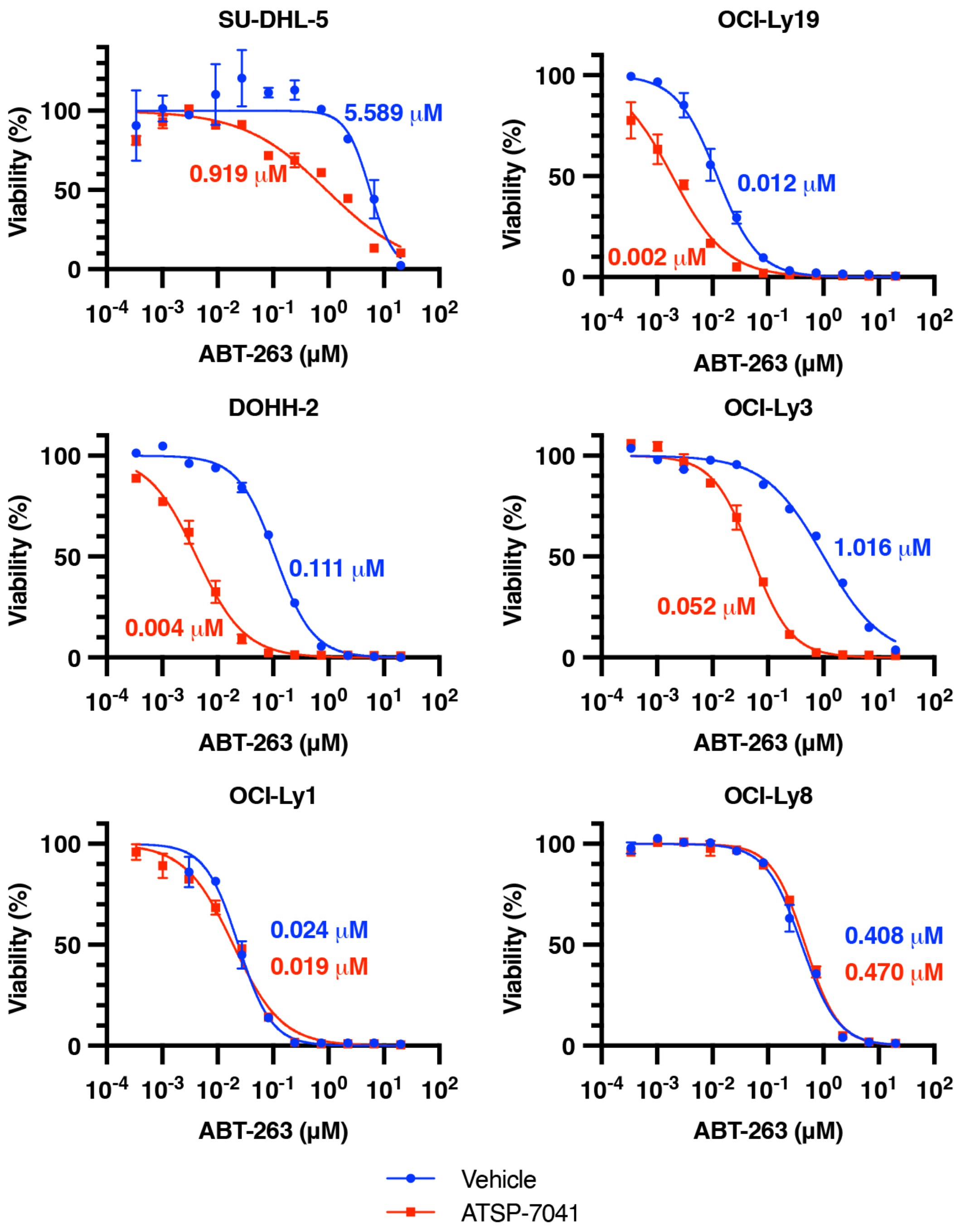

Figure S3: Cell death sensitivity to ABT-263 after priming with ATSP-7041. Cells were treated for 24 hr with ATSP-7041 or vehicle control. Pre-treated cells were then washed and plated with dose titrations of ABT-263, and viability was measured 24 hr later. Data were normalized to untreated cells from the same pre-treatment condition, plotted as the mean of duplicates ± SEM, and fitted using nonlinear regression. EC_{50} is shown.

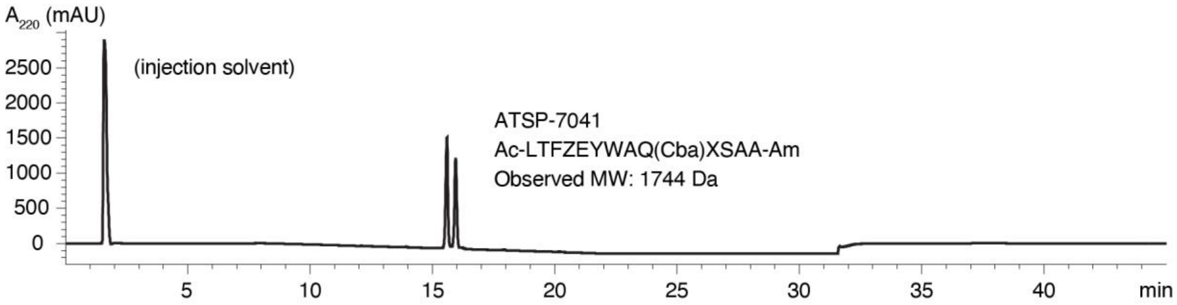

Figure S4: LCMS analysis of purified ATSP-7041. Peptide purity was confirmed by LCMS, integrating the area under the A_{220} curve. Peptide purity was > 95% for each batch. ATSP-7041 eluted as two peaks due to the staple existing as two isomers, as previously reported.[71]

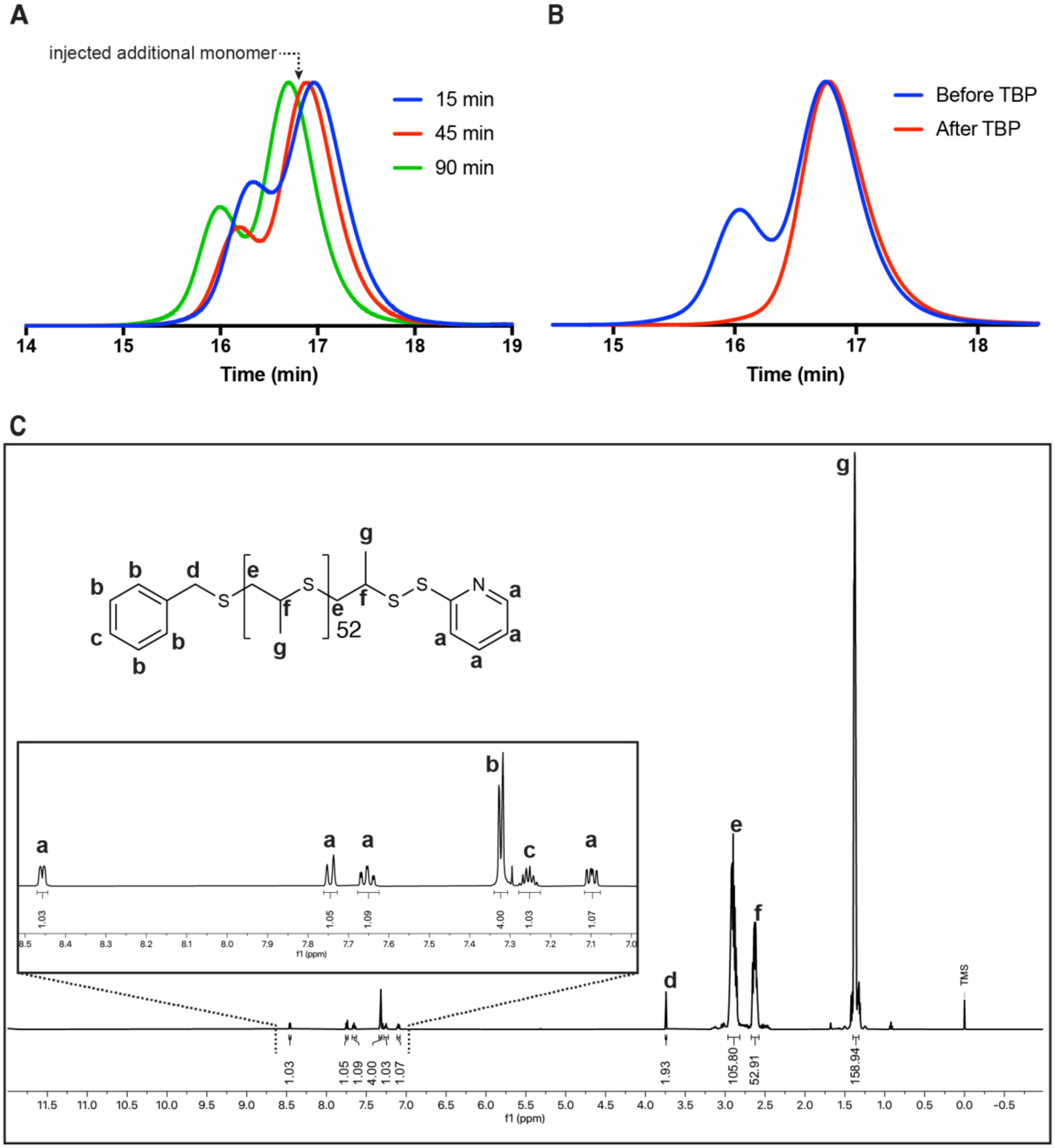

Figure S5: Characterization of PPS-PDS. (A) During PPS-SH polymerization, two peaks were visible by GPC with mobile phase DMF + 0.01 M LiBr at 50 °C, and both peaks grew to larger MW with additional monomer. The larger MW polymer (leftmost peak) was presumably two PPS-SH polymers linked by a disulfide bond. **(B)** Reduction with TBP eliminated the higher MW peak, reducing disulfide polymers back into PPS-SH. The dispersity of reduced PPS-SH was 1.17. **(C)** ^{1}H NMR spectrum of PPS-PDS in $CDCl_3$.

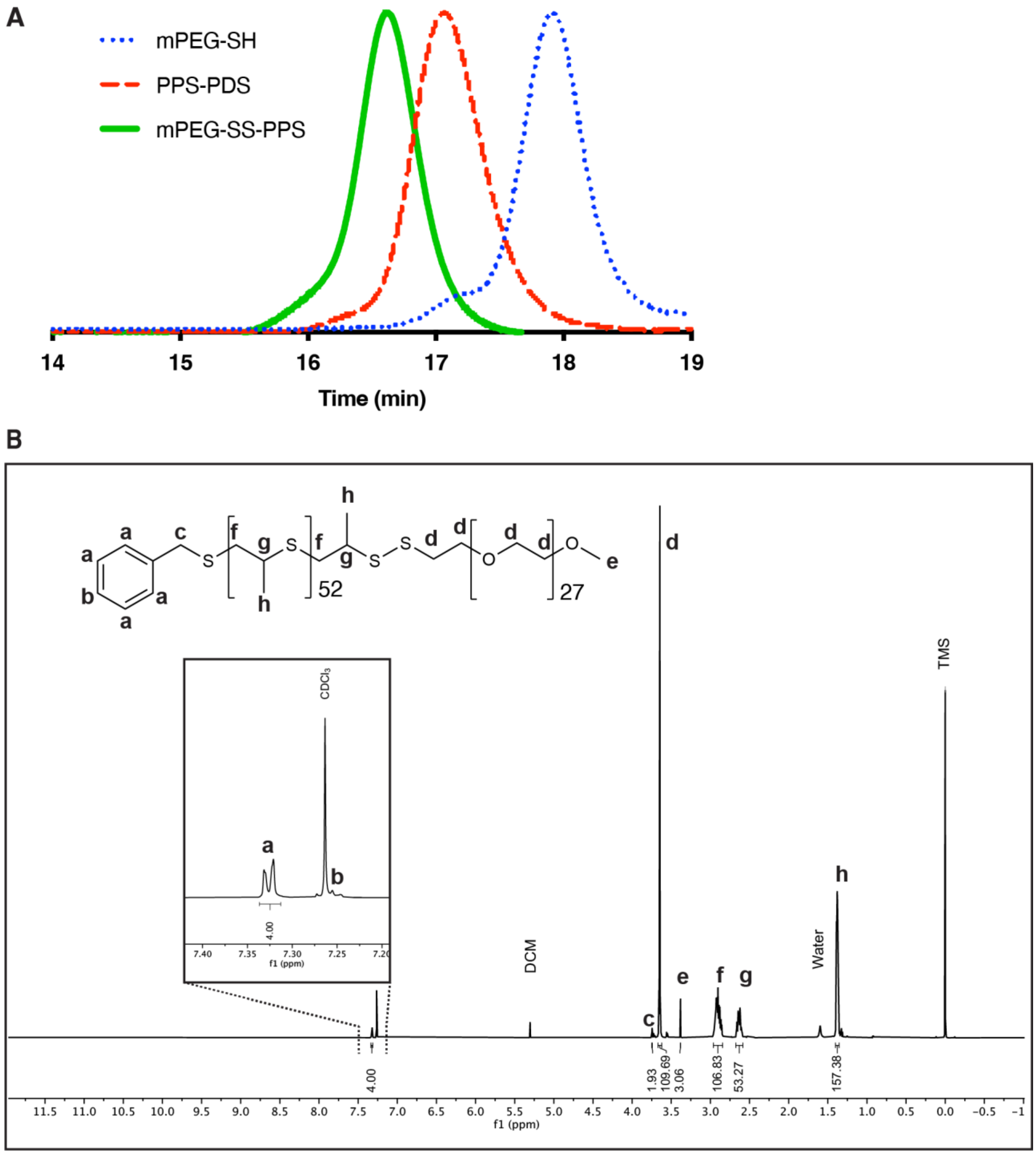

Figure S6: Characterization of mPEG-SS-PPS. (A) Chromatogram of mPEG-SS-PPS (Dispersity = 1.08) and its precursor polymers measuring refractive index via GPC with mobile phase DMF + 0.01 M LiBr at 50 °C. **(B)** ^{1}H NMR spectrum of mPEG-SS-PPS in $CDCl_3$.

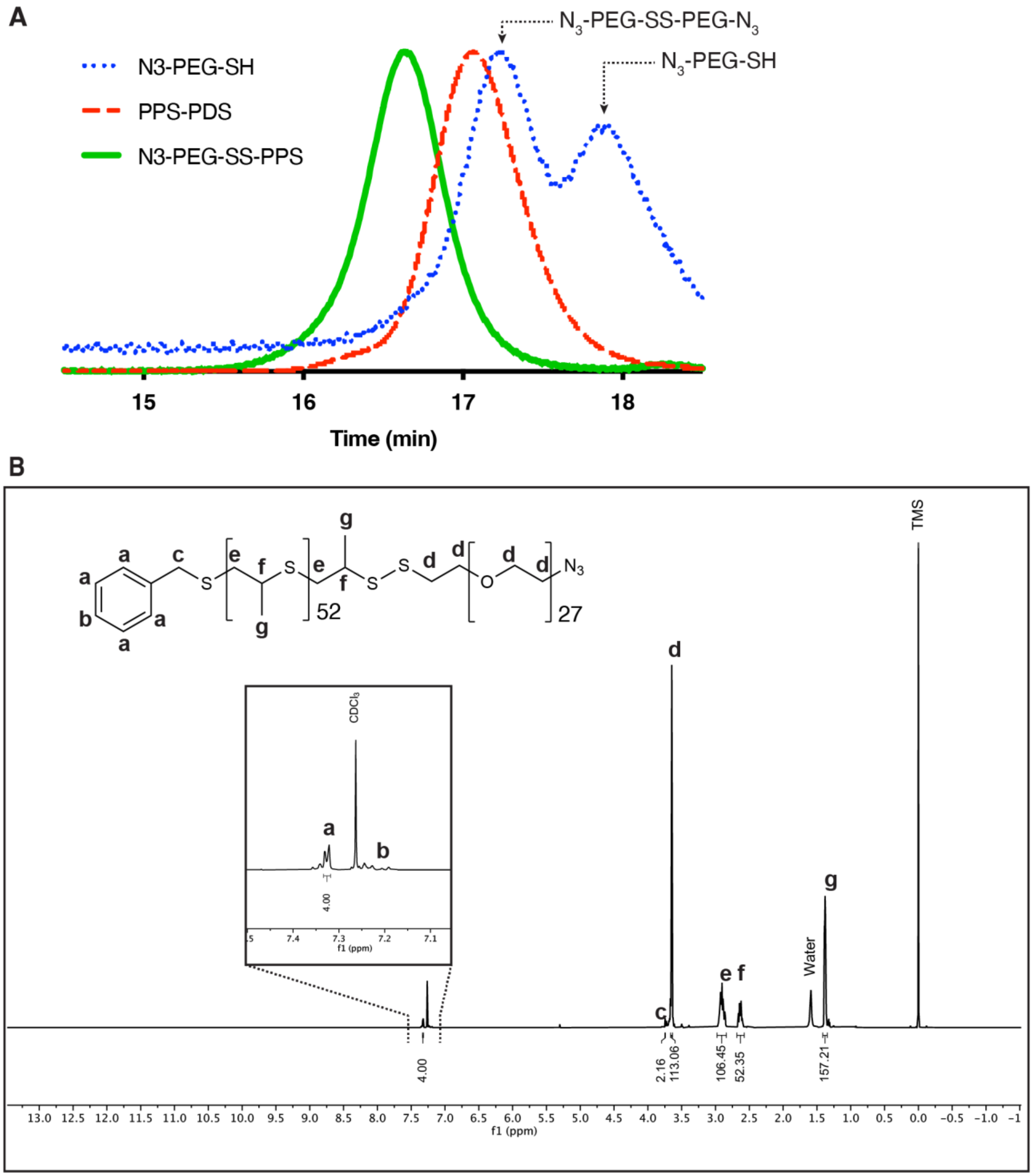

Figure S7: Characterization of N_3-PEG-SS-PPS. (A) Chromatogram of N_3-PEG-SS-PPS (Dispersity = 1.06) and its precursor polymers measuring refractive index via GPC with mobile phase DMF + 0.01 M LiBr at 50 °C. **(B)** ^{1}H NMR spectrum of N_3-PEG-SS-PPS in $CDCl_3$.

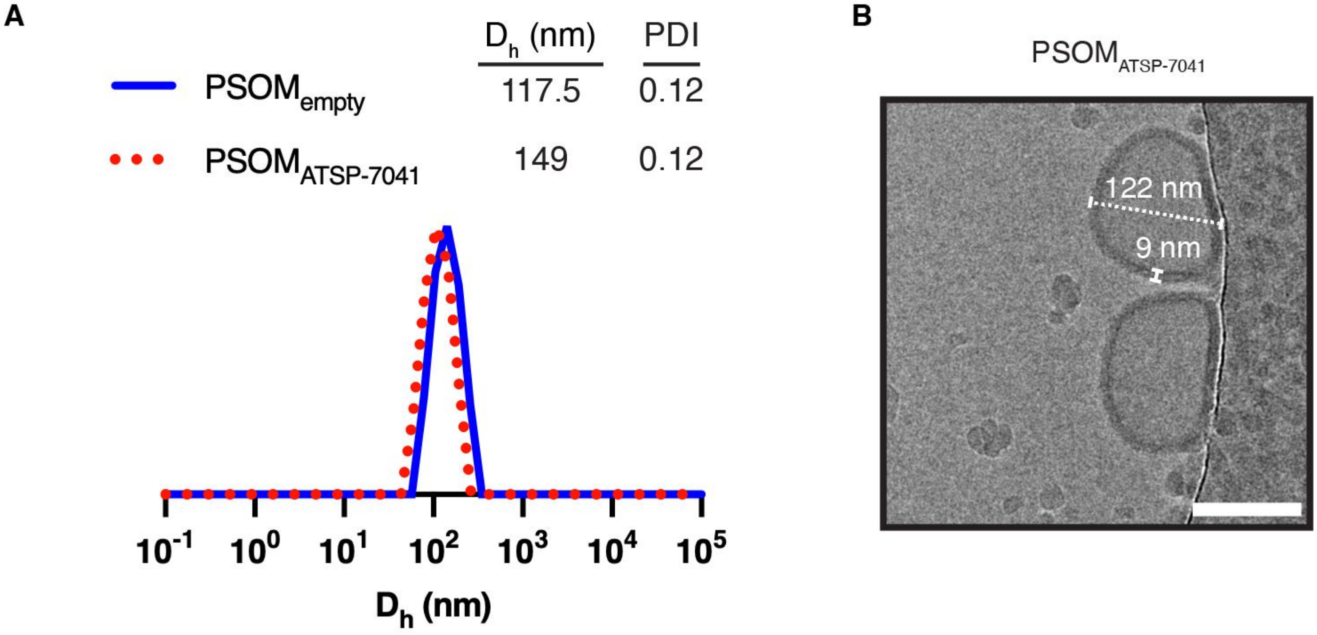

Figure S8: Characterization of $PSOM_{ATSP-7041}$. (A) DLS measurements of $PSOM_{empty}$ and $PSOM_{ATSP-7041}$ show similar hydrodynamic diameter (D_h). **(B)** Cryo-TEM confirms that $PSOM_{ATSP-7041}$ forms hollow spheres with diameter and bilayer thickness similar to $PSOM_{empty}$ (Figure 3J). Scale bar is 100 nm.

Region	Antibody	Positions	Sequence	Positions	Translation
V_k	αCD19	1 - 30	GATATCTTGCTCACCCAAACTCCAGCTTCT	1 - 10	DILLTQTPAS
		31 - 60	TTGGCTGTGTCTCTAGGGCAGAGGGCCACC	11 - 20	LAVSLGQRAT
		61 - 90	ATCTCCTGCAAGGCCAGCCAAAGTGTTGAT	21 - 30	ISCKASQSVD
		91 - 120	TATGATGGTGATAGTTATTTGAACTGGTAT	31 - 40	YDGDSYLNWY
		121 - 150	CAACAGATTCCAGGACAGCCACCCAAACTC	41 - 50	QQIPGQPPKL
		151 - 180	CTCATCTATGATGCATCCAATCTAGTTTCT	51 - 60	LIYDASNLVS
		181 - 210	GGAATCCCACCCAGGTTTAGTGGCAGTGGG	61 - 70	GIPPRFSGSG
		211 - 240	TCTGGGACAGACTTCACCCTCAACATCCAT	71 - 80	SGTDFTLNIH
		241 - 270	CCTGTGGAGAAGGTGGATGCTGCAACCTAT	81 - 90	PVEKVDAATY
		271 - 300	CACTGTCAGCAAAGTACTGAGGACCCGTGG	91 - 100	HCQQSTEDPW
		301 - 330	ACGTTCGGTGGAGGCACCAAGCTGGAAATC	101 - 110	TFGGGTKLEI
		331 - 333	AAA	111	K
	αOspA	1 - 30	GATATTCAAATGACCCAGAGTCCAAGTAGT	1 - 10	DIQMTQSPSS
		31 - 60	CTTAGTGCCACACTGGGCGGTAAAGTGACG	11 - 20	LSATLGGKVT
		61 - 90	ATCACTTGTAAAGCCAGCCAGGACATCAAC	21 - 30	ITCKASQDIN
		91 - 120	AAATACATAGCTTGGTATCAGCATAAACCG	31 - 40	KYIAWYQHKP
		121 - 150	GGAAAGGGACCGAGACTGCTGATCCATTAT	41 - 50	GKGPRLLIHY
		151 - 180	ACTTCCACGCTCCAGCCCGGTAACCCAAGC	51 - 60	TSTLQPGNPS
		181 - 210	CGATTTTCTGGGAGCGGAAGTGGTAGGGAT	61 - 70	RFSGSGSGRD
		211 - 240	TATAGCTTTTCCATTAGCAACTTGGAAGCC	71 - 80	YSFSISNLEA
		241 - 270	GAAGACATTGCTATATACTACTGTCTGCAA	81 - 90	EDIAIYYCLQ
		271 - 300	TATGACAACTTGCAGCGCACTTTTGGTGGG	91 - 100	YDNLQRTFGG
		301 - 321	GGAACCAAAGTGGAAATTAAG	101 - 107	GTKVEIK
V_H	αCD19	1 - 30	CAAGTGCAACTCCAACAGTCAGGGGCTGAG	1 - 10	QVQLQQSGAE
		31 - 60	CTGGTGAGGCCTGGCTCCTCAGTGAAGATT	11 - 20	LVRPGSSVKI
		61 - 90	TCCTGCAAGGCTTCTGGCTATGCATTCAGT	21 - 30	SCKASGYAFS
		91 - 120	AGCTACTGGATGAACTGGGTGAAGCAGAGG	31 - 40	SYWMNWVKQR
		121 - 150	CCTGGACAGGGTCTTGAGTGGATTGGACAG	41 - 50	PGQGLEWIGQ
		151 - 180	ATTTGGCCTGGAGATGGTGATACTAACTAC	51 - 60	IWPGDGDTNY
		181 - 210	AATGGAAAGTTCAAGGGTAAAGCCACTCTG	61 - 70	NGKFKGKATL
		211 - 240	ACTGCAGACGAATCCTCCAGCACAGCCTAC	71 - 80	TADESSSTAY
		241 - 270	ATGCAACTCAGCAGCCTAGCATCTGAGGAC	81 - 90	MQLSSLASED
		271 - 300	TCTGCGGTCTATTTCTGTGCAAGACGGGAG	91 - 100	SAVYFCARRE
		301 - 330	ACTACGACGGTAGGCCGTTATTACTATGCT	101 - 110	TTTVGRYYYA
		331 - 360	ATGGACTACTGGGGTCAAGGAACCTCAGTC	111 - 120	MDYWGQGTSV
		361 - 372	ACCGTCTCGAGC	121-124	TVSS
	αOspA	1 - 30	CAGATCCAACTCGTGCAGTCTGGACCCGAG	1 - 10	QIQLVQSGPE
		31 - 60	TTGAAGAAACCCGGTGAGACTGTAAAGATT	11 - 20	LKKPGETVKI
		61 - 90	TCTTGCAAAGCCTCCGGCTACACATTTACC	21 - 30	SCKASGYTFT
		91 - 120	GATTATTCCATGTACTGGGTGAAGCAGGCA	31 - 40	DYSMYWVKQA
		121 - 150	CCTGGAAAAGGGTTGAAAAGGATGGGTTGG	41 - 50	PGKGLKRMGW
		151 - 180	ATAAACACAGAAACCGGCGAACCAACCTAT	51 - 60	INTETGEPTY
		181 - 210	GCCGACGACTTCAAGGGTCGATTCGCGCTT	61 - 70	ADDFKGRFAL
		211 - 240	TCCCTGGATACTTCCGCCTCCACCGCATAC	71 - 80	SLDTSASTAY
		241 - 270	CTTCACATTTCCAACCTCAAGAACGAAGAC	81 - 90	LHISNLKNED
		271 - 300	ACAGCTACATACTTTTGCGCCCGAGGACTT	91 - 100	TATYFCARGL
		301 - 330	GATAGCTGGGGTCAGGGTACTAGTGTTACC	101 - 110	DSWGQGTSVT
		331 - 339	GTCTCGAGC	111 - 113	VSS
C_k		1 - 30	CGGGCTGATGCGGCGCCAACTGTATCCATC	1 - 10	RADAAPTVSI
		31 - 60	TTCCCACCATCCAGTGAGCAGTTAACATCT	11 - 20	FPPSSEQLTS
		61 - 90	GGAGGTGCCTCAGTCGTGTGCTTCTTGAAC	21 - 30	GGASVVCFLN
		91 - 120	AACTTCTACCCCAAAGACATCAATGTCAAG	31 - 40	NFYPKDINVK
		121 - 150	TGGAAGATTGATGGCAGTGAACGACAAAAT	41 - 50	WKIDGSERQN
		151 - 180	GGCGTCCTGAACAGTTGGACTGATCAGGAC	51 - 60	GVLNSWTDQD
		181 - 210	AGCAAAGACAGCACCTACAGCATGAGCAGC	61 - 70	SKDSTYSMSS
		211 - 240	ACCCTCACGTTGACCAAGGACGAGTATGAA	71 - 80	TLTLTKDEYE
		241 - 270	CGACATAACAGCTATACCTGTGAGGCCACT	81 - 90	RHNSYTCEAT
		271 - 300	CACAAGACATCAACTTCACCCATTGTCAAG	91 - 100	HKTSTSPIVK
		301 - 321	AGCTTCAACAGAAATGAGTGT	101 - 107	SFNRNEC
C_H		1 - 30	GCCAAAACGACACCCCCATCTGTCTATCCA	1 - 10	AKTTPPSVYP
		31 - 60	CTGGCCCCTGGATCTGCTGCCCAAACTAAC	11 - 20	LAPGSAAQTN
		61 - 90	TCCATGGTGACCCTGGGATGCCTGGTCAAG	21 - 30	SMVTLGCLVK
		91 - 120	GGCTATTTCCCTGAGCCAGTGACAGTGACC	31 - 40	GYFPEPVTVT
		121 - 150	TGGAACTCTGGGTCCCTGTCCAGCGGTGTG	41 - 50	WNSGSLSSGV
		151 - 180	CACACCTTCCCAGCTGTCCTGCAGTCTGAC	51 - 60	HTFPAVLQSD
		181 - 210	CTCTACACTCTGAGCAGCTCAGTGACTGTC	61 - 70	LYTLSSSVTV
		211 - 240	CCCTCCAGCACCTGGCCCAGCGAGACCGTC	71 - 80	PSSTWPSETV
		241 - 270	ACCTGCAACGTTGCCCACCCGGCCAGCAGC	81 - 90	TCNVAHPASS
		271 - 300	ACCAAGGTGGACAAGAAAATTGTGCCCAGG	91 - 100	TKVDKKIVPR
		301 - 312	GATTGCGGATCC	101 - 104	DCGS
Cysteine Linker		1 - 24	GGCGGTTCCAGCGGCAGCGGCTGC	1 - 8	GGSSGSGC

Figure S9: DNA coding sequences of αCD19 and αOspA Fabs and their protein translations. Fabs were designed with variable domains ($V_κ$ and V_H) for binding to either human CD19 (αCD19) or a xenoantigen control (αOspA). All Fabs shared the same constant domains ($C_κ$ and C_H). For αCD19-cys and αOspA-cys, the cysteine linker sequence was added to the C-terminal end of the C_H domain.

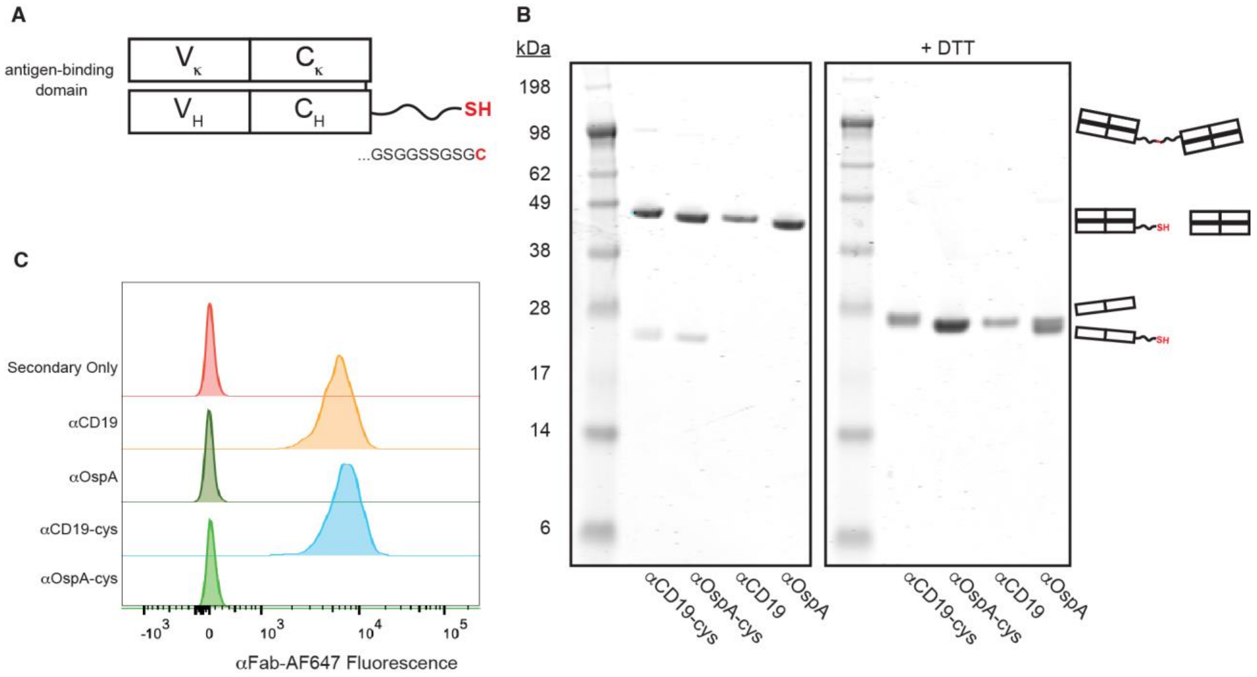

Figure S10: Expression and binding validation of αCD19 and αOspA Fabs. (A) Fabs were designed using previously published sequences from antibodies that bind either human CD19 (αCD19) or a xenoantigen control (αOspA) as described in Materials and Methods.[73-76] To enable site-specific conjugation to polymersomes, a flexible cysteine linker was encoded at the C-terminus of the heavy chain of each Fab to generate αCD19-cys and αOspA-cys. **(B)** Coomassie-stained SDS-PAGE of purified Fabs. Each Fab appears pure at the expected molecular weights. To confirm proper Fab formation, addition of DTT in the loading buffer reduces the interchain disulfide to generate polypeptides (heavy chain and light chain) that overlap at their expected molecular weights. **(C)** Flow cytometric measurement of Fab binding to a $CD19^+$ DLBCL cell line, SU-DHL-5. Cells were stained with the indicated Fab, followed by an AF647-labeled αFab secondary antibody. αCD19 Fabs bind $CD19^+$ DLBCL with or without the cysteine linker, and the control (αOspA) Fabs do not.

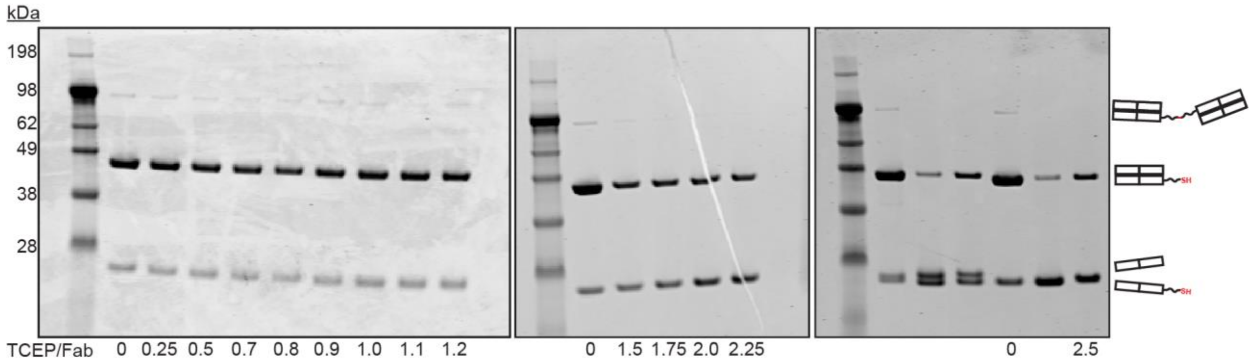

Figure S11: Full Coomassie-stained SDS-PAGE gels from Figure 4C. αOspA-cys was reduced with varying amounts of TCEP, reacted with Sulfo-DBCO-PEG4-Maleimide, purified by desalting, and separated on SDS-PAGE gels. The gels were Coomassie-stained, and the bands in each lane were quantified using ImageJ. Unlabeled lanes contain samples irrelevant to this work.

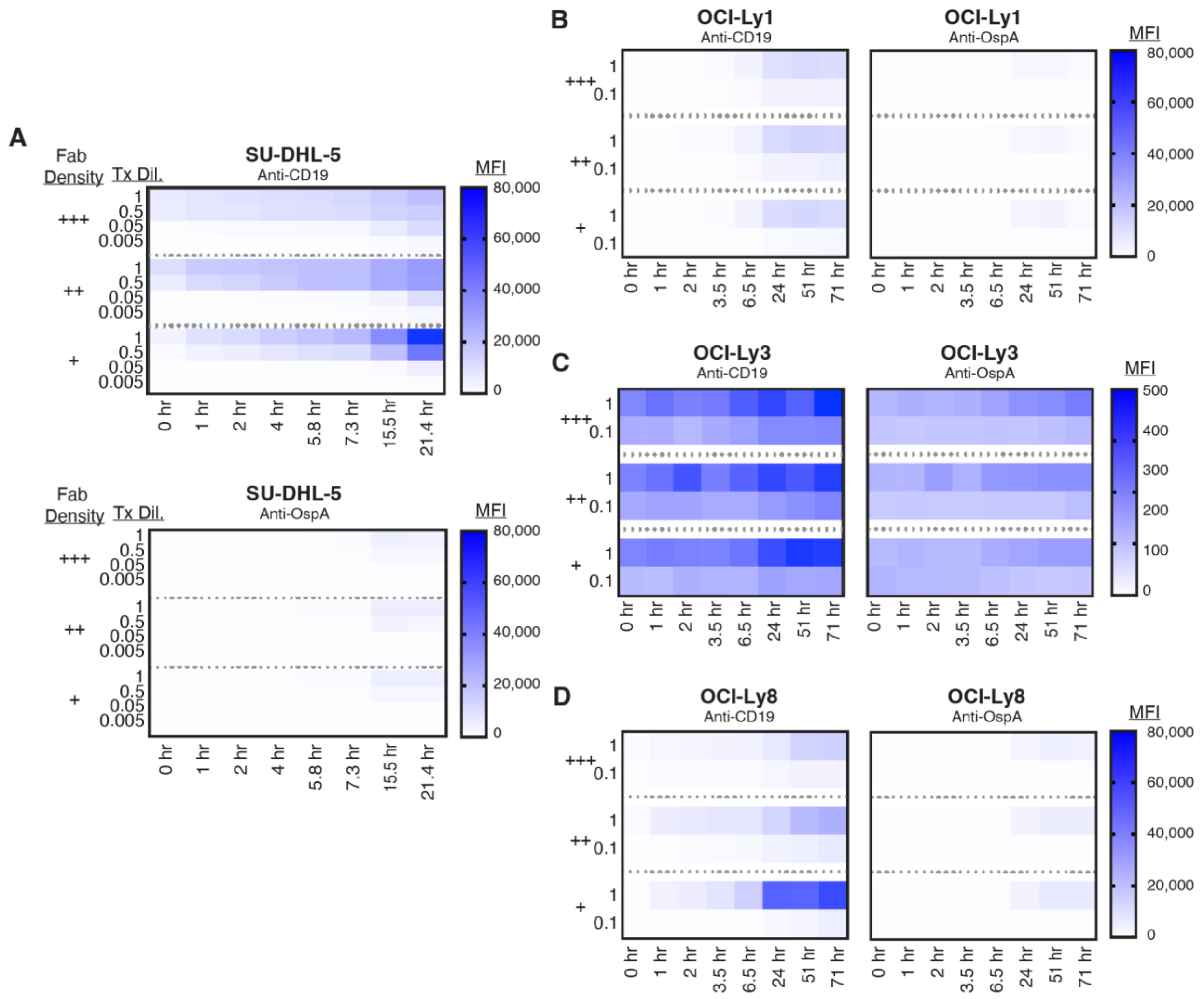

Figure S12: CD19 targeting enhances polymersome delivery of calcein into various DLBCL cell lines. DLBCL cells were treated with CD19-targeted or xenoantigen (OspA) targeted polymersomes loaded with calcein and analyzed by flow cytometry. Treatment concentrations were held constant across samples based on calcein absorbance after Triton X-100 disruption and calcein dequenching. The uptake of fluorescent polymersomes was dependent upon time, concentration, CD19 targeting, and Fab density. αCD19 Fab functionalization greatly improved cellular uptake, and lower Fab densities caused more uptake. This was conducted in **(A)** SU-DHL-5, **(B)** OCI-Ly1, **(C)** OCI-Ly3, and **(D)** OCI-Ly8. The data in (D) are identical to Figure 4E, shown for comparison across cell lines. +++ = 1% theoretical Fab density, ++ = 0.5% theoretical Fab density, + = 0.1% theoretical Fab density.

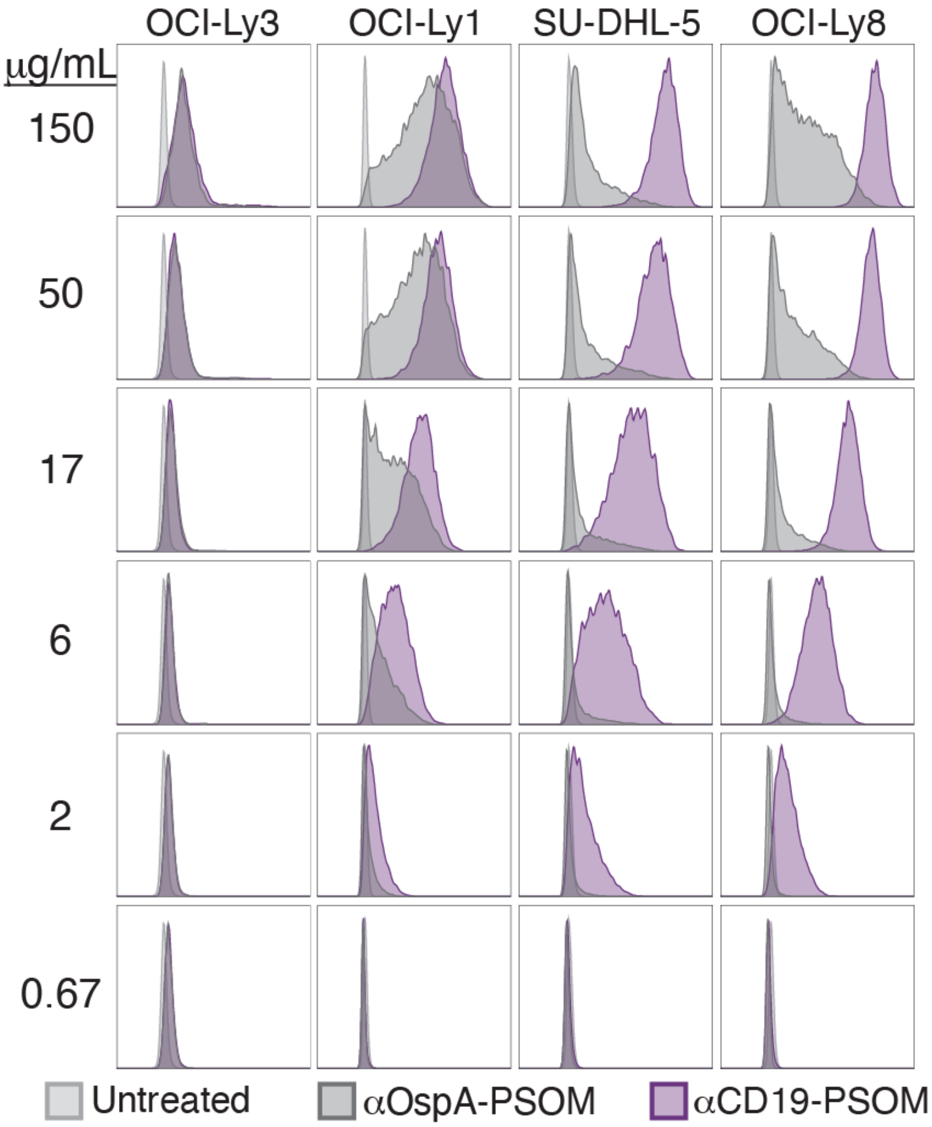

Figure S13: Polymersome uptake into DLBCL cell lines depends upon CD19 cell surface expression and polymersome dose. DLBCL cell lines were treated with calcein-loaded polymersomes for 24 hr and then analyzed by flow cytometry. Both specific uptake/binding (αCD19; purple) and non-specific uptake/binding (αOspA; dark gray) were dose-dependent, but CD19-mediated uptake was significantly greater. The total polymer concentration in each treatment is indicated in µg/mL.

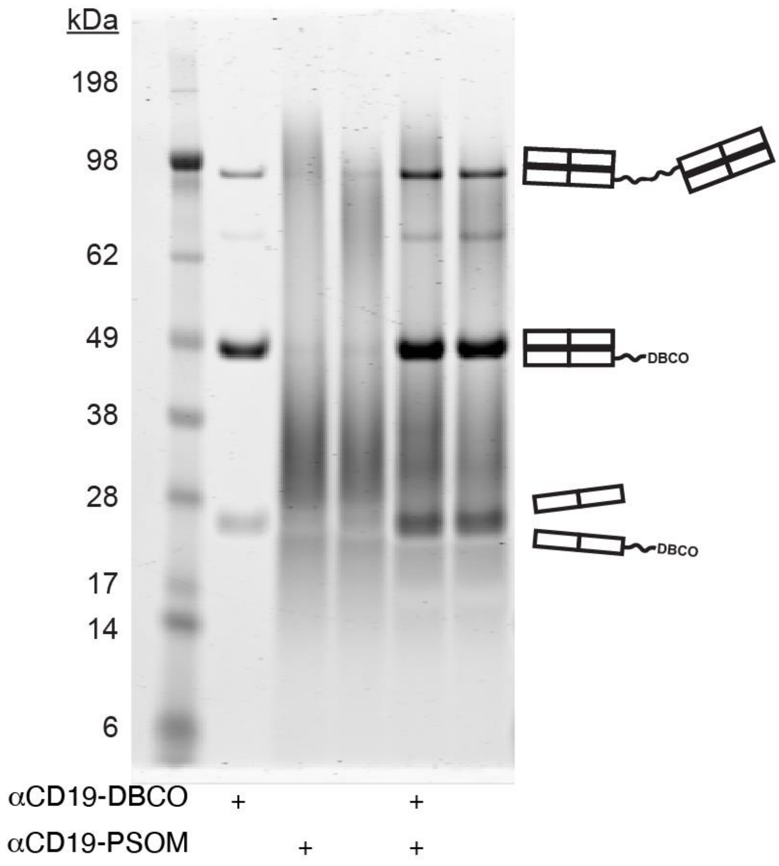

Figure S14: Full Coomassie-stained SDS-PAGE gel from Figure 4H. Bands from Figure 4H are in labeled lanes, and unlabeled lanes contain samples irrelevant to this work. All gel samples were prepared with sodium azide (to quench DBCO:azide reactions) and NEM (to quench thiols and disulfide shuffling). Broad polymer smearing occurred in the presence of sodium azide in the gel samples. Gel samples were loaded such that, assuming 100% Fab conjugation, the Fab bands would be identical. This was calculated using the GPC-measured polymer concentration.

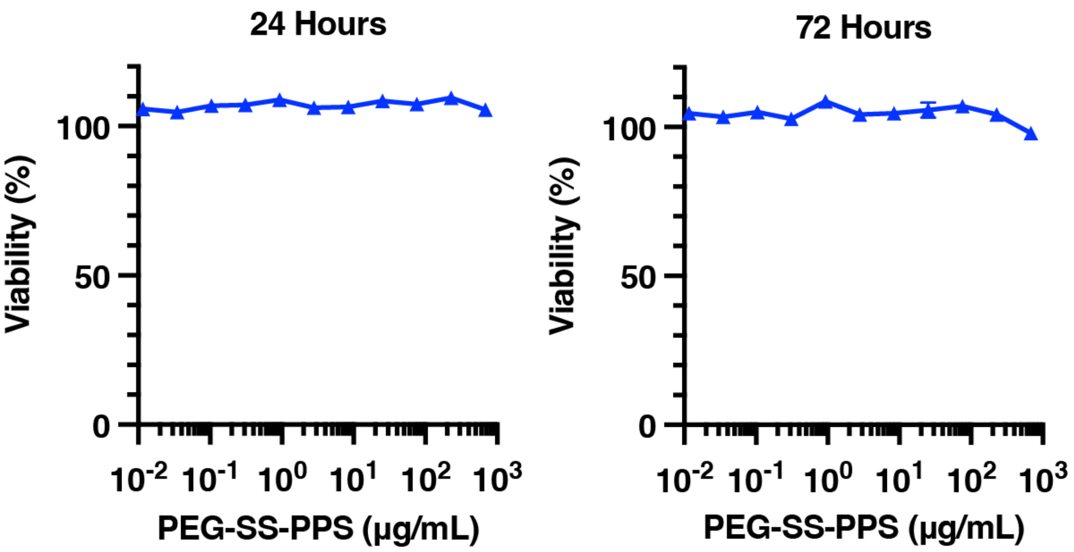

Figure S15: PEG-SS-PPS is non-toxic at high concentrations. OCI-Ly19 cells were treated with αCD19-PSOM$_{empty}$ at varying concentrations of PEG-SS-PPS as high as 682 µg/mL for 24 hr and 72 hr. No concentration was achieved that inhibited cell viability. Viability was measured using CellTiter-Glo 2.0, normalized to untreated cells, and plotted as the mean of duplicates ± SEM.

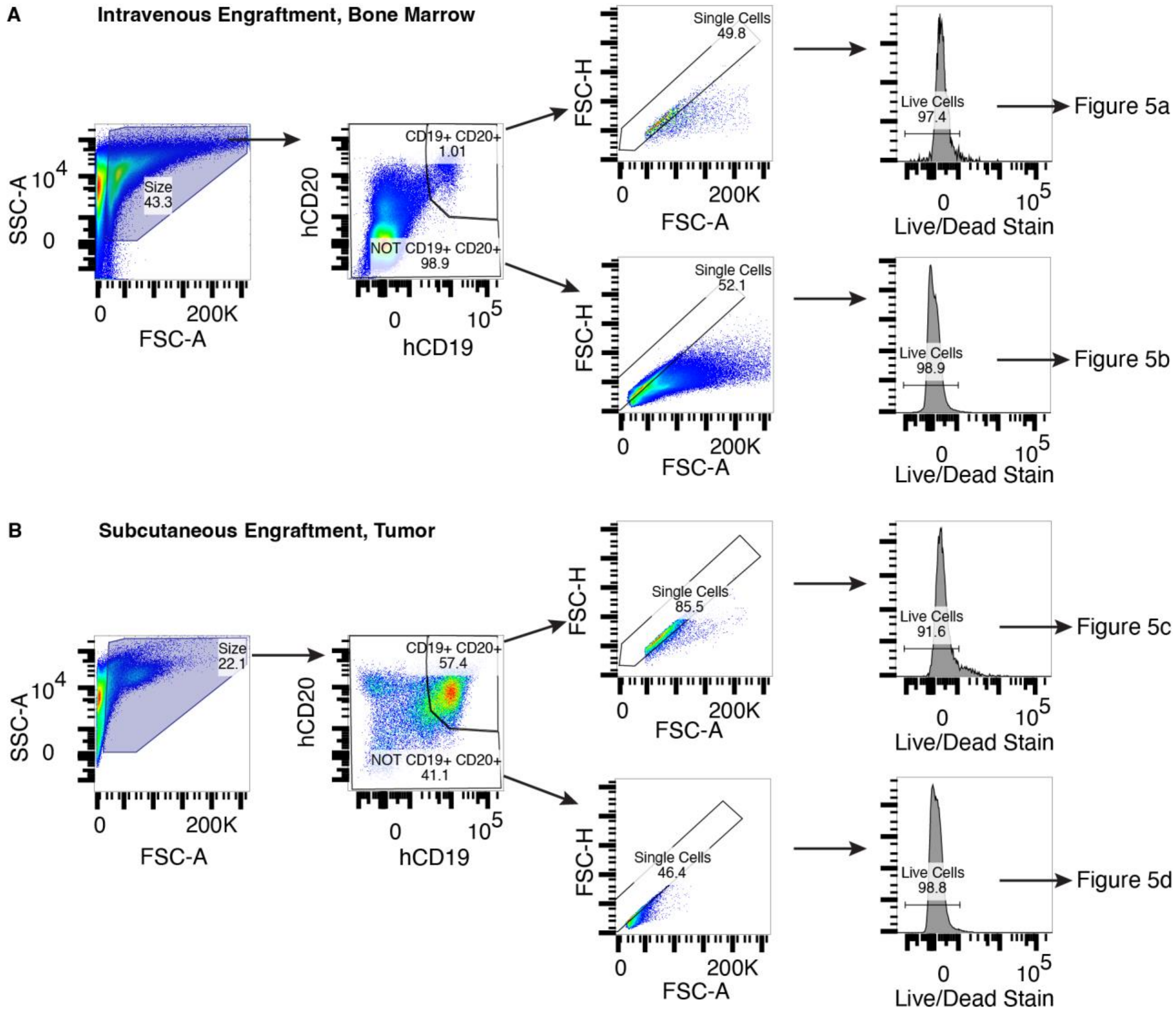

Figure S16: Representative gating strategy for flow cytometry of DLBCL cells from xenografts. After the treatment in Figure 5, **(A)** bone marrow and **(B)** subcutaneous tumors were isolated as single-cell suspensions and stained for flow cytometry. Debris was excluded via gating of FSC-A versus SSC-A. DLBCL cells ($CD19^{+}CD20^{+}$) were distinguished from other cells in the tissue. The indicated live cell populations were used in Figure 5 to measure fluorescence from mice treated with vehicle or αCD19-$PSOM_{calcein}$.

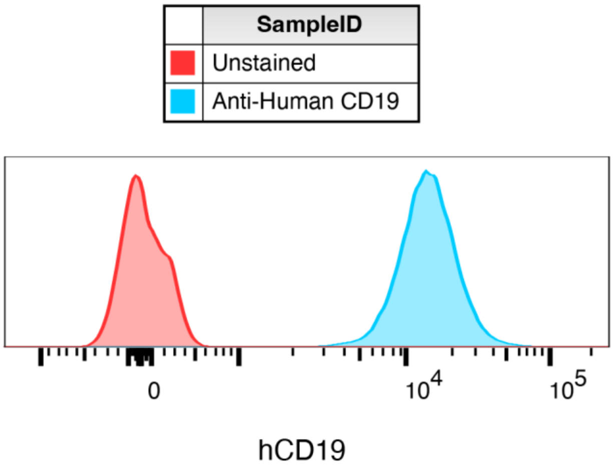

Figure S17: OCI-Ly19 expresses CD19. CD19 expression was measured using flow cytometry after staining cells with an APC-conjugated anti-human-CD19 antibody.

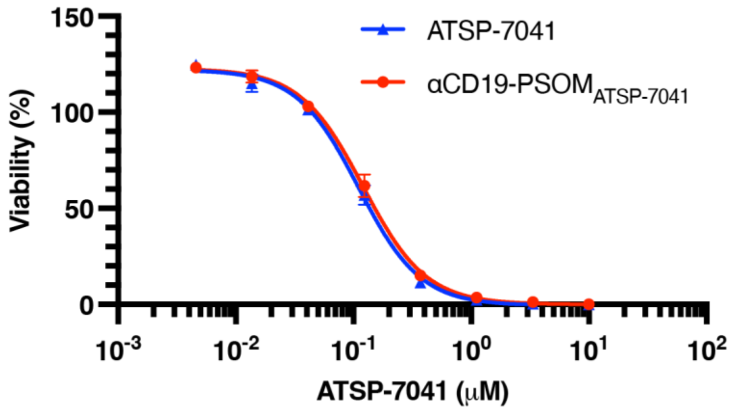

Figure S18: Polymersome delivery maintains the efficacy of ATSP-7041. OCI-Ly19 cells were treated with free ATSP-7041 (blue) or αCD19-PSOM$_{ATSP-7041}$ (red) at varying concentrations of ATSP-7041 for 72 hr. Viability was normalized to untreated cells and plotted as the mean of duplicates ± SEM.
